# Converged Rab37/IL-6 trafficking and STAT3/PD-1 transcription axes elicit an immunosuppressive lung tumor microenvironment

**DOI:** 10.7150/thno.60040

**Published:** 2021-05-12

**Authors:** I-Ying Kuo, You-En Yang, Pei-Shan Yang, Yu-Jou Tsai, Hong-Tai Tzeng, Hung-Chi Cheng, Wan-Ting Kuo, Wu-Chou Su, Chih-Peng Chang, Yi-Ching Wang

**Affiliations:** 1 Department of Pharmacology, College of Medicine, National Cheng Kung University, Tainan, Taiwan.; 2 Department of Biochemistry, College of Medicine, National Cheng Kung University, Tainan, Taiwan College of Medicine, National Cheng Kung University, Tainan, Taiwan.; 3 Institute of Basic Medical Sciences, College of Medicine, National Cheng Kung University, Tainan, Taiwan.; 4 Division of Oncology, Department of Internal Medicine, College of Medicine, National Cheng Kung University, Tainan, Taiwan.; 5 Department of Microbiology and Immunology, College of Medicine, National Cheng Kung University, Tainan, Taiwan.

**Keywords:** Rab37, IL-6, PD-1, transcription, tumor microenvironment

## Abstract

**Background:** Increased IL-6 level, M2 macrophages and PD-1^+^CD8^+^ T cells in tumor microenvironments (TME) have been identified to correlate with resistance to checkpoint blockade immunotherapy, yet the mechanism remains poorly understood. Rab small GTPase-mediated trafficking of cytokines is critical in immuno-modulation. We have previously reported dysregulation of Rab37 in lung cancer cells, whereas the roles of Rab37 in tumor-infiltrating immune cells and cancer immunotherapy are unclear.

**Methods:** The tumor growth of the syngeneic mouse allograft in wild type or *Rab37* knockout mice was analyzed. Imaging analyses and vesicle isolation were conducted to determine Rab37-mediated IL-6 secretion. STAT3 binding sites at *PD-1* promoter in T cells were identified by chromatin immunoprecipitation assay. Multiplex fluorescence immunohistochemistry was performed to detect the protein level of Rab37, IL-6 and PD-1 and localization of the tumor-infiltrating immune cells in allografts from mice or tumor specimens from lung cancer patients.

**Results:** We revealed that Rab37 regulates the secretion of IL-6 in a GTPase-dependent manner in macrophages to trigger M2 polarization. Macrophage-derived IL-6 promotes STAT3-dependent *PD-1* mRNA expression in CD8^+^ T cells. Clinically, tumors with high stromal Rab37 and IL-6 expression coincide with tumor infiltrating M2-macrophages and PD1^+^CD8^+^ T cells that predicts poor prognosis in lung cancer patients. In addition, lung cancer patients with an increase in plasma IL-6 level are found to be associated with immunotherapeutic resistance. Importantly, combined blockade of IL-6 and CTLA-4 improves survival of tumor-bearing mice by reducing infiltration of PD1^+^CD8^+^ T cells and M2 macrophages in TME.

**Conclusions:** Rab37/IL-6 trafficking pathway links with IL-6/STAT3/PD-1 transcription regulation to foster an immunosuppressive TME and combined IL-6/CTLA-4 blockade therapy exerts potent anti-tumor efficacy.

## Introduction

Immune cells are known to accumulate in tumor microenvironment (TME) and crucial for regulating tumor progression 1, 2. Up till now, there are more than 20 immune checkpoints found, and the blockade therapies targeting immune inhibitory receptors such as cytotoxic T lymphocyte associated protein-4 (CTLA-4) and programmed cell-death receptor 1 (PD-1) or its ligand, programed cell death protein ligand 1 (PD-L1) are now front-line treatments 3-6. Nevertheless, many patients show primary or acquired resistance to immune checkpoint inhibitors 7, 8. For example, increased density of CD11b^+^F4/80^+^ or CD163^+^ tumor associated macrophages (TAMs) has been identified to closely correlate with such resistance 9, 10. Further, enrichment of exhausted PD-1^+^CD8^+^ T cells in melanoma patients correlates with poor response to immunotherapy 11. The mechanism that induces these potent immunosuppressive cells or exhausted PD-1^+^CD8^+^ T cells in TME is not completely clear. In particular, how TME regulates PD-1 upregulation on CD8^+^ T cells is still a puzzle.

Elevated IL-6 level is linked to chronic inflammatory conditions and observed in many types of cancers 12. Increased IL-6 in TME will trigger hyperactivation of downstream JAK/STAT3 signaling to drive proliferation, angiogenesis, and metastasis of tumor cells 13-16. Notably, the increase of IL-6 associated pathways has been found to enhance M2-TAMs and exhausted T cell populations in TME 9, 17. Although it is known that tumor-infiltrating immune cells are a primary source of IL-6 production in TME, the mechanism of IL-6 secretion is not fully understood.

Rabs are a large group of small GTPases and function as regulators of vesicle trafficking and protein transport. The activity of Rab proteins is controlled by active GTP-bound and inactive GDP-bound forms. Dysregulation of Rab GTPases has been reported to associate with tumorigenesis. We have previously reported that Rab37 mediates exocytosis of several secretion factors in non-small cell lung (NSCLC) cells 18, 19. In addition, Rab37 facilitates soluble IL-1 receptor-like protein secretion in NSCLC cells to antagonize IL-33-mediated M2 macrophage polarization in TME 20. To date, the role of Rab37 in tumor-infiltrating immune cells remains unknown.

Here, we report that Rab37-mediated exocytosis of IL-6 in NSCLC-associated macrophages promotes their M2 polarization and further PD-1 upregulation in T cells* via* the Rab37/IL-6/STAT3 transcription axis. Combined blockade of IL-6 and CTLA-4 significantly suppresses lung tumor growth and metastasis in mice. Clinically, NSCLC patients with increased intratumoral Rab37^+^IL-6^+^ immune cells where enriched with tumor infiltrated CD163^+^ M2-TAMs and exhausted PD-1^+^CD8^+^ T cells show poor prognosis. Our findings highlight the crucial role of Rab37/IL-6/PD-1 axis in supporting immunosuppressive TME.

## Materials and Methods

### Generation of *Rab37* knockout (KO) mice

The specific mouse *Rab37* KO target vector is shown in Figure S1A. The *Rab37* gene was deleted before injecting into C57BL/6J (B6) mice using *Cre-loxP* system to establish the whole body depleted *Rab37* KO mice. Embryonic stem cells were selected by neomycin and genotyped by Southern blotting. The selected cells were injected into blastocysts to generate chimeric mice later used for producing *Rab37* KO mice. Mouse studies were approved by the Institutional Animal Care and Use Committee of National Cheng Kung University (NCKU) (Permit Numbers: #106255).

### Cytokine array

Rab37 positive-related cargo proteins in supernatants of BMDMs derived from wild-type (WT) and *Rab37* KO mice were detected with mouse cytokines antibody array-membrane (96 targets) (#ab193659, Abcam, Cambridge, MA, US). Media derived from BMDMs of WT mice and *Rab37* KO mice treated with the conditioned media (CM) from mouse Lewis lung carcinoma (LLC) cells were collected at 0 h (MOCK) or 24 h (LCM). The array was performed according to the manufacturer's instructions.

### Vesicle isolation and chromatin immunoprecipitation (ChIP) assays

RAW264.7 cells (2.5×10^6^) were sonicated and supernatants were obtained by centrifugation (3,000 *g* for 10 min at 4 °C) and vesicles were enriched from supernatants by high speed centrifugation (30,000 *g* for 60 min at 4 °C) using a 40-Ti rotor (Beckman, Duarte, CA, US). The vesicles-containing solution (500 μg) was incubated with anti-V5 antibody to isolate Rab37-specific vesicles and the IL-6 cargos in vesicles were analyzed by Western blot (Table S2). For ChIP assay, Jurkat T cells (5×10^6^) were cross-linked to prepare nuclear lysates using Magna ChIP^TM^ protein G Kit (Millipore, Cork, Ireland) followed by immunoprecipitation with 5 μg anti-p-STAT3(Y705) antibody (#9145, Cell Signaling, Danvers, MA, US). Q-PCR was carried out using ChIP products. The primer sequences are listed in Table S3.

### Allograft tumor growth and metastasis assays* in vivo*

All animal experiments were performed in compliance with NCKU institutional guidelines for use and care of animals (Permit Numbers: #106255). For α-IL-6 (#MP5-20F3; BioXCell, Lebanon, New Hampshire, US) and α-CTLA-4 (#9H10; BioXCell) treatment, LLC subcutaneous injection and orthotopic injection models were used. For subcutaneous model, 5×10^5^ LLC cells were subcutaneously injected into flank of WT or *Rab37* KO B6 mice. 1×10^4^ LLC cells were suspended in 100 μl PBS for orothotopic model. In both models, 250 μg of IgG, α-IL-6 and/or α-CTLA-4 were given by intraperitoneal injection in B6 mice harboring LLC allograft. For subcutaneous model, tumor volume was calculated at day 6 and once every five days using the equation V = (a^2^ x b)/2 during observation. For orthotopic model, mice were sacrificed at day 21 and the primary injected lung and contralateral lungs were removed and resected for RNA/protein extraction, flow cytometry (FLOW) analysis and preserved in paraffin for immunofluorescence immunohistochemistry (IF-IHC) staining. Blood samples were collected for serum biochemistry examinations for toxicity.

### Patient samples and clinical information

A total of 62 surgically resected lung cancer patients were recruited from NCKU Hospital, after obtaining appropriate institutional review board permission (#B-ER-106-102) and informed consent from the patients. Overall survival was calculated from the day of surgery to the date of death or the last follow-up. Tumor typing and disease staging were performed according to the World Health Organization classification and the TNM classification system, respectively. A total of 16 lung cancer patients receiving clinical α-PD-1 Ab treatment were also recruited (#B-ER-106-102). All patients received regular follow-up at 3-month intervals to evaluate the treatment responses. Partial responders showed no enlargement or newly developed distant metastatic foci on CT scan, while poor non-responders were patients with enlargement or newly developed distant metastatic foci on CT scan. Blood samples were collected at pre-treatment and 3-month on-treatment of α-PD-1 therapy for ELISA analysis. The detailed clinicopathological characteristics of patients are listed in Table S4.

### Statistical analysis

Cell studies were conducted in three independent experiments unless indicated otherwise. The number of mouse per group for animal studies was described for each experiment. Two-tailed Student's t test was used. Data represented mean ± SD. Pearson χ^2^ test was used to compare the correlation of Rab37, IL-6, CD45, CD163, CD8 or PD-1 protein expression in lung cancer patients. Overall survival curves were calculated according to the Kaplan-Meier method, and comparison was performed using the log-rank test. Cox regression comparison was performed to analyze the relative risk for patient poor outcome. The level of statistical significance was taken as p value, *, p < 0.05; **, p < 0.01; ***, p < 0.001.

Detailed methods are enclosed in the online supplemental materials and methods section.

## Results

### *Rab37*-/- KO mice provide an anti-tumor microenvironment to inhibit tumor growth

**Figure 1:**
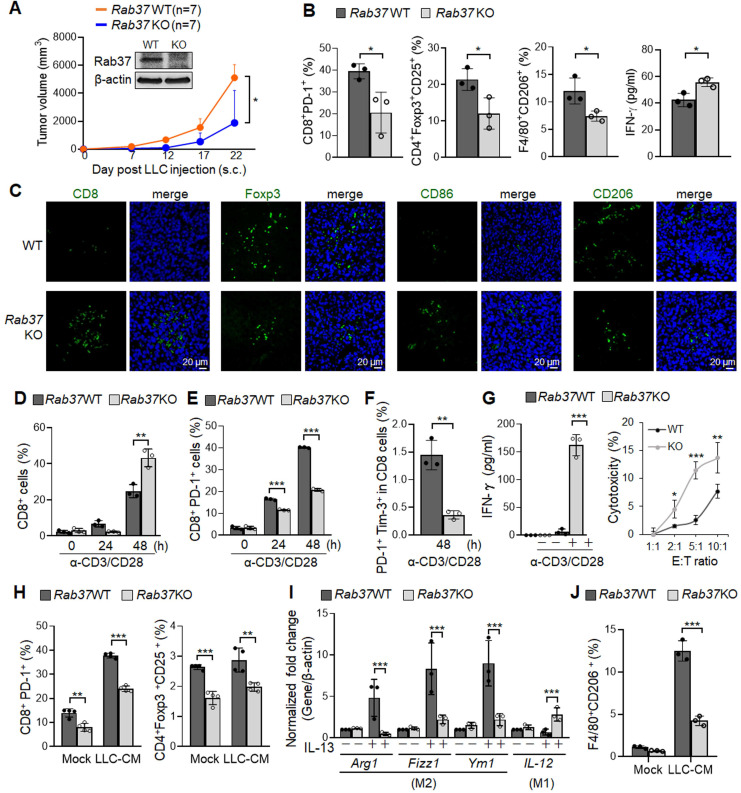
** Rab37 in stromal immune cells links immunosuppression to tumor-promoting phenotypes. (A)** Tumor growth of the subcutaneous syngeneic mouse LLC allograft (1 x 10^6^ cells inoculated) in *Rab37* WT and KO C57BL/6 mice. Immunoblots of Rab37 expression are shown. **(B, C)** Tumor-infiltrating T cells and macrophages of endpoint tumor on day 22 were analyzed by FLOW (B) or IF-IHC (C). Scale bars: 20 µm. **(D-F)** Total CD8^+^ cells (D), PD-1^+^CD8^+^ cells (E), and PD-1^+^Tim-3^+^CD8^+^ cells (F) of isolated splenocytes from tumor-free *Rab37* WT or KO mice upon stimulation by anti-CD3 and anti-CD28 antibodies for 48 h. **(G)** *Left*: IFN-γ secretion on splenic CD8^+^ T cells from tumor-free *Rab37* WT or KO mice upon anti-CD3/anti-CD28 stimulation, *Right*: WT and *Rab37* KO mouse effector splenocytes (E) were co-cultured with Luciferase-transduced mouse LLC cancer target cells (T) at various E:T ratios for 48 h to determine the cytotoxicity against cancer cells. **(H)** Analysis of PD-1 expression on CD8^+^ T (*left*) and Treg population of CD4^+^ T (*right*) splenocytes upon *ex vivo* treatment with LLC-CM (conditioned media). **(I)** mRNA level of *Arg1, Fizz1, Ym1* (M2) and *IL-12* (M1) by RT-qPCR after M2 stimulator IL-13 treatment in isolated* Rab37* WT and KO BMDMs. **(J)** Analyses of percentage of F4/80^+^CD206^+^ by FLOW after LLC-CM treated* Rab37* WT and KO BMDMs. Data represent mean ± SD. * p < 0.05; ** p < 0.01; *** p < 0.001, Student's *t*-test.

To understand how Rab37 expression in the stromal cells impacts lung cancer progression, we generated whole-body *Rab37* KO mice in C57BL/6 background (Figure S1A). Rab37 deficiency was confirmed at mRNA and protein levels (Figure 1A, S1B). We then observed for tumor growth of the subcutaneous syngeneic mouse LLC allograft model in WT and *Rab37* KO mice. The results showed that LLC tumor growth in *Rab37* KO mice was inhibited compared to that in WT mice (Figure 1A). We further examined physiological relevance of *Rab37* KO in tumor metastasis using tail-vein injection model. The size and number of metastatic cancer nodules in lungs were lower in* Rab37* KO mice than in WT mice (Figure S1C-E). These results suggested that depletion of stromal Rab37 created an anti-tumor microenvironment in the primary site and in metastatic niches.

Next, we collected endpoint subcutaneous LLC allografts to analyze tumor-infiltrating T cells and macrophages by FLOW analysis (Figure 1B). Tumor-infiltrating CD8^+^ T cells derived from *Rab37* KO mice showed reduced PD-1 expression compared to those from WT mice, indicating Rab37 involvement in PD-1 expression on CD8^+^ T cells. FLOW analyses further revealed that the allografts derived from *Rab37* KO had decreased number of Tregs and CD206^+^ M2 macrophages compared to WT mice (Figure 1B). The IF-IHC results confirmed that Rab37 deficiency increased CD8^+^ T cells and M1 macrophages, but decreased Tregs and M2 macrophages in the TME of LLC primary allografts (Figure 1C) and metastatic lung tumor sections (Figure S1F). These findings uncovered a pro-tumor role of stromal-residing Rab37 in fostering an immunosuppressive TME.

### Rab37 in stromal immune cells links immunosuppression to tumor-promoting phenotypes

Next, we investigated the contribution of Rab37 in endogenous immune response to LLC tumor. We first isolated splenocytes from *Rab37* WT and KO mice and stimulated them with anti-CD3/CD28 antibodies. FLOW analysis results showed that the percentage of CD8^+^ T cells from *Rab37* KO mice was increased as compared to *Rab37* WT mice upon anti-CD3/anti-CD28 stimulation (Figure 1D). Moreover, CD8^+^ T cells from* Rab37* KO mice had significantly reduced surface PD-1 (Figure 1E). Importantly, exhausted CD8^+^ T cell subpopulation (PD-1^+^Tim-3^+^CD8^+^) was significantly lower in T cells from *Rab37* KO mice compared with WT mice (Figure 1F). In addition, the ELISA results showed an increase of IFN-γ secretion upon anti-CD3/anti-CD28 stimulation (*Left*, Figure 1G) and the lymphocyte-mediated cytotoxicity against cancer cell assay demonstrated an enhanced cancer cell killing effect of CD8^+^ T cells from *Rab37* KO mice (*Right*, Figure 1G). The isolated splenic CD8^+^ T cells were then treated with LLC conditioned medium (LLC-CM). LLC-CM treatment increased the PD-1 expression on WT CD8^+^ T cells compared to those from *Rab37* KO mice (*Left*, Figure 1H). Moreover, the amount of Treg was also decreased in *Rab37* KO splenocytes compared to WT cells upon LLC-CM treatment, although no significant differences were observed in LCC-CM and MOCK groups of WT Tregs due to the lack of T cell receptor signaling upon LLC-CM treatment (*Right*, Figure 1H). Together, these results revealed that splenic T cells of *Rab37* KO mice had enhanced proliferation, cytokine production and cytotoxicity against cancer cells.

We further examined the role of Rab37 in macrophages by isolating BMDMs from WT and *Rab37* KO mice. BMDMs were then induced towards M2 polarization by stimulating with IL-13. RT-qPCR results showed that *Rab37* KO BMDMs displayed reduced expression of M2 associated genes (*Arg1, Fizz1* and *Ym1*) as compared to WT cells. Conversely, *Rab37* KO BMDMs showed enhanced expression of M1 marker *IL-12* gene even upon IL-13 stimulation (Figure 1I). Notably, FLOW data indicated that LLC-CM treatment induced M2 surface marker CD206 on WT BMDMs in response to LLC-CM treatment (Figure 1J). Together, these findings reveal previously unrecognized functions of Rab37 in promoting pro-tumorigenic M2 type macrophage polarization and limiting cytotoxic T cell activity.

### Cytokine/chemokine array and ELISA identify cargo candidates of Rab37 in macrophages

**Figure 2:**
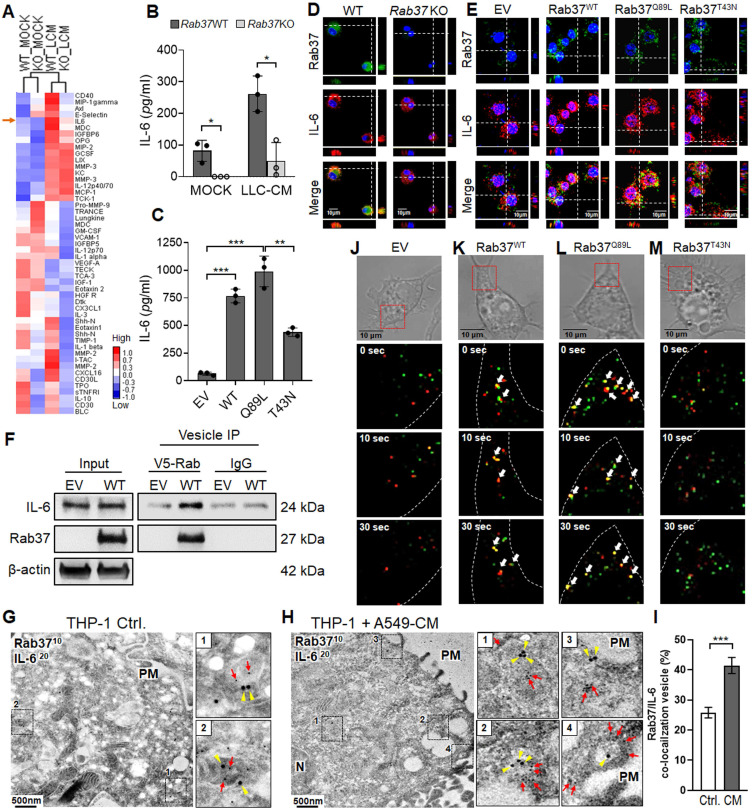
** Rab37 mediates IL-6 secretion in macrophages in a GTPase-dependent manner. (A)** Heatmap depicting the secretin level of cytokines/proteins in CM derived from untreated or LLC-CM treated BMDMs from *Rab37* WT and KO mice. IL-6 is indicated by an arrow. **(B, C)** ELISA was performed to validate the level of IL-6 in CM from *Rab37* WT and KO BMDMs (b) or in CM from EV, Rab37^WT^, Rab37^Q89L^ or Rab37^T43N^ RAW264.7 cells (C). **(D, E)** Confocal microscopy images of Rab37 (green), IL-6 (red) and nucleus staining (blue) in *Rab37* WT and KO BMDMs (D) or in EV, Rab37^WT^, Rab37^Q89L^ or Rab37^T43N^ RAW264.7 cells (E). Z-stack images are shown. Scale bars: 10 µm. **(F)** Vesicles of EV or Rab37^WT^ RAW264.7 cells expressing V5-tagged Rab37, were collected by centrifugations and immunoprecipitated (IP) with anti-V5 and vesicle lysates were blotted for V5-Rab37 and endogenous IL-6.** (G-I)** Ultrastructural localization of Rab37 (10 nm of gold, red arrow) and IL-6 (20 nm of gold, yellow triangle) illustrated by immuno-EM images of control THP-1 (G) or A549-CM treated THP-1 macrophages (H). PM: plasma membrane. Scale bars: 500 nm. Enlarged images shown in insets of the representative regions. (I) Rab37/IL-6 colocalized vesicles were significantly increased in the A549-CM treated THP-1 compared to those in the control THP-1 cells. An average of 15-25 vesicles per cell and a total of 10 cells were collected. Percentages are expressed in relation to the total number of Rab37 specific vesicles. **(J-M)** Selected frames from time-lapse confocal movies of GFP-tagged EV, Rab37^WT^, Rab37^Q89L^, or Rab37^T43N^ RAW264.7 cells co-transfected with RFP-tagged IL-6 cells. Enlarged images of the boxed areas with time intervals in seconds are shown (bottom). Arrow indicates trafficking vesicle. Scale bars: 10 µm. Data represent mean ± SD. * p < 0.05; ** p < 0.01; *** p < 0.001, Student's *t*-test.

Several exocytic Rab small GTPases have been reported to target the release of immune modulating proteins to shape the TME 21. Thus far, TNFα is the putative cytokine cargo identified for Rab37 in macrophage and T cells 22, 23. To identify the potential cargos of Rab37 in macrophages, one of the major immune cells to infiltrate TME, LLC-CM educated WT and *Rab37* KO BMDMs were collected and subjected to cytokine/chemokine array analyses (Figure 2A). Our data demonstrated that the secretion levels of several cytokines and chemokines including IL-6 were decreased in *Rab37* KO BMDMs compared with WT BMDMs at basal level or upon LLC-CM stimulation. The increase of IL-6 associated pathways has been found to enhance M2-TAMs and inactivated T cell populations in TME 9, 17. Therefore, we selected IL-6 as target candidate of Rab37-mediated exocytosis and explored the impact of IL-6 modulated pathways in fostering an immunosuppressive TME.

ELISA assay confirmed that the secretion levels of IL-6 were decreased in LLC-CM educated *Rab37* KO BMDMs compared to WT BMDMs (Figure 2B). Since Rab37 is a small GTPase regulated by GTP or GDP, we expressed empty vector control (EV), Rab37 wild-type (Rab37^WT^), constitutively active Rab37-GTP mutant (Rab37^Q89L^) and dominant negative Rab37-GDP mutant (Rab37^T43N^) in RAW264.7 murine macrophage cell line. ELISA data showed that IL-6 level was elevated in CM from Rab37^WT^ and Rab37^Q89L^, but reduced in Rab37^T43N^ RAW264.7 cells (Figure 2C). These data suggest that Rab37 mediates IL-6 secretion in macrophages in a GTPase-dependent manner.

### IL-6 is a trafficking cargo of Rab37 in macrophages

To investigate whether Rab37 directly regulates exocytosis of IL-6 in macrophages, we conducted confocal analysis to demonstrate that Rab37 co-localized with IL-6 in WT BMDMs (*Left*, Figure 2D), while the co-localization signals disappeared in *Rab37* KO BMDMs (*Right*, Figure 2D). In addition, Rab37 co-localized with IL-6 to form yellow fluorescent in Rab37^WT^ and Rab37^Q89L^ cells, whereas Rab37 and IL-6 were distributed to distinct regions in the Rab37^T43N^ cells (Figure 2E).

Next, to demonstrate the presence of IL-6 in the Rab37-containing vesicles, we overexpressed V5-tagged EV and Rab37^WT^ in RAW264.7 cells and performed vesicles isolation to immunoprecipitate Rab37-specific vesicles with anti-V5 beads. The vesicle IP-western blots showed that IL-6 was enriched in Rab37-specific vesicles isolated from Rab37^WT^ RAW264.7 cells (Figure 2F). To further confirm the ultrastructure of Rab37-mediated vesicle recruitment of IL-6, we performed immuno-transmission electron microscopy on cancer CM treated THP-1 human monocyte cells. Strikingly, IL-6 was localized in Rab37-associated vesicles in THP-1 cells (Figure 2G, I), which was further enhanced by cancer-CM (Figure 2H-I). Colocalization of IL-6 with Rab37-containing vesicles was increased in close proximity of plasma membrane (PM) in the cancer-CM treated THP-1 monocytes (*Regions 3 and 4*, Figure 2H). Moreover, IL-6 colocalized with Rab37 in the WT BMDMs (Figure S2A), but not in *Rab37* KO BMDMs (Figure S2B). Collectively, these results demonstrate that IL-6 localizes in Rab37-containing vesicles in macrophages.

For real-time visualization of Rab37-regulated IL-6 trafficking, we performed time-lapse confocal microscopic analysis of GFP-tagged EV, Rab37^WT^, Rab37^Q89L^, or Rab37^T43N^ RAW264.7 macrophage cells co-transfected with RFP-tagged IL-6 expression vector (Figure 2J-M, Movie S1-S4). In Rab37^WT^ RAW264.7 cells, IL-6 (red) was progressively taken up into Rab37 (green) punctate vesicular structures to form colocalization vesicles (yellow) and then rapidly released for another run of Rab37-mediated trafficking (Figure 2K). In Rab37^Q89L^ RAW264.7 cells, more colocalized puncta and faster trafficking were observed as compared to Rab37^WT^ cells (Figure 2L). In Rab37^T43N^ RAW264.7 cells, Rab37 and IL-6 punctate structures appeared in distinct compartment (Figure 2M). We also employed TIRF microscopy analysis for selective visualization of fluorescently labeled vesicles located in close proximity to the PM (Figure S2C-E, Movie S5-S6). To the best of our knowledge, this is the first report on the dynamics of Rab37-mediated trafficking and docking of IL-6-containing vesicles.

### Rab37-mediated IL-6 secretion promotes M2 macrophage polarization *via* STAT3 activation

**Figure 3:**
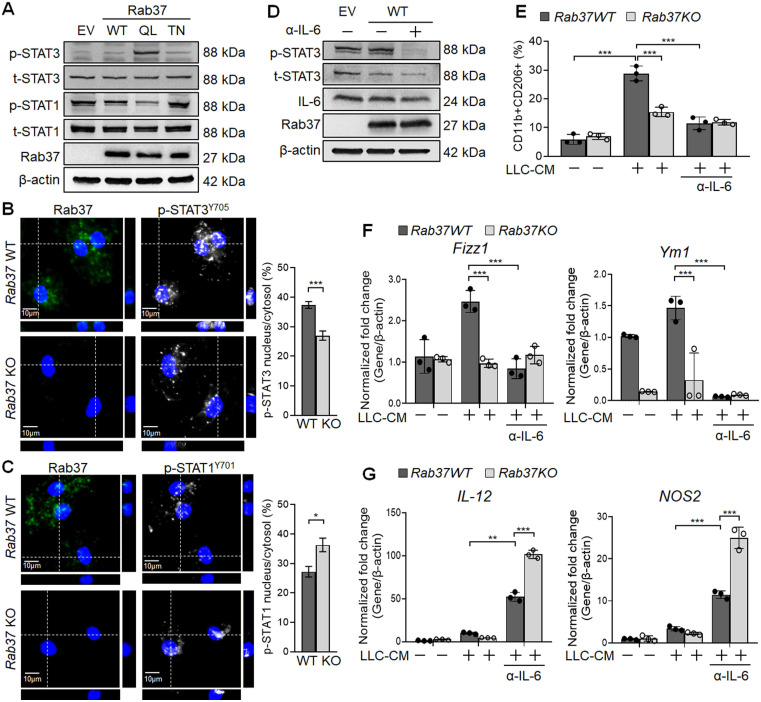
** Rab37 mediates IL-6 secretion to promote M2 macrophage polarization *via* STAT3 activation and STAT1 down-regulation. (A)** Immunoblots of p-STAT3 and p-STAT1 in RAW264.7 cells transfected with EV, Rab37^WT^, Rab37^Q89L^ and Rab37^T43N^.** (B, C)** IF images (*Left*) and quantification (*Right*) of p-STAT3 translocation to nucleus in *Rab37* WT BMDMs compared to KO BMDMs (B) while p-STAT1 showed more nuclear translocation in *Rab37* KO BMDMs than in WT BMDMs (C). Z-stack images are shown. Scale bars: 10 µm. **(D, E)** Treatment of α-IL-6 antibody (α-IL-6) down-regulated p-STAT3 in Rab37^WT^ RAW264.7 cells (D) or M2 marker (CD11b^+^CD206^+^) in* Rab37* WT BMDMs (E). **(F, G)** RT-qPCR analysis was performed to examine the mRNA expression of M2 genes *Fizz1* and* Ym1* (F) and M1 genes *IL-12* and* NOS2* (G) in BMDMs from *Rab37* WT and KO mice treated with LLC-CM and/or α-IL-6 for 24 h. Treatment of α-IL-6 reduced expression of the M2 genes but up-regulated the M1 genes. Data represent mean ± SD. ** p < 0.01; *** p < 0.001, Student's *t*-test.

Previous studies suggested that IL-6 promotes M2 polarization by activating STAT3 24. Yet, STAT1 activation drives M1 macrophage polarization 25. This prompted us to examine the STAT3 and STAT1 activation in EV, Rab37^WT^, Rab37^Q89L^ and Rab37^T43N^ expressing RAW264.7 cells. Western blot data showed that p-STAT3 level was elevated in Rab37^Q89L^ RAW264.7 cells whereas p-STAT1 level was down-regulated in Rab37^WT^ and Rab37^Q89L^ RAW264.7 cells (Figure 3A). Additionally, IF results demonstrated that nuclear localization of p-STAT3 was enhanced in WT BMDMs comparing to *Rab37* KO BMDMs (*Upper* and *Right*, Figure 3B), while decreased nuclear localization of p-STAT1 was observed in WT BMDMs (*Upper* and *Right*, Figure 3C). We further performed reconstitution analyses in Rab37^WT^ cell treated with neutralizing IL-6 antibody (α-IL-6). The immunoblotting results showed that treatment of α-IL-6 attenuated Rab37^WT^-induced p-STAT3 signals (Figure 3D). These results support the notion that Rab37 mediates IL-6 secretion in macrophages to up-regulate STAT3 and down-regulate STAT1 transcription program.

To explore the role Rab37 in promoting M2-polarization, we treated BMDMs isolated from WT or *Rab37* KO mice with LLC-CM. FLOW analysis demonstrated that LLC-CM treatment efficiently induced M2 polarization in *Rab37* WT BMDMs (F4/80^+^CD206^+^), which was reduced by α-IL-6 treatment (Figure 3E). Notably, α-IL-6 greatly reduced the mRNA level of M2 marker genes (*Fizz1* and *Ym1*) and induced the M1 genes (*IL-12* and *NOS2*) in BMDMs (Figure 3F-G). The LLC-CM induced M2 polarization in *Rab37*-WT BMDMs was reduced by α-IL-6 treatment (Figure 3F-G). Together, these results suggest that Rab37 mediates IL-6 secretion to skew M2 macrophage polarization by enhancing STAT3 but reducing STAT1 signal.

### Macrophage-derived IL-6 promotes PD-1 expression in T cells *via* the Rab37/IL-6/STAT3 trafficking and transcription axes

**Figure 4:**
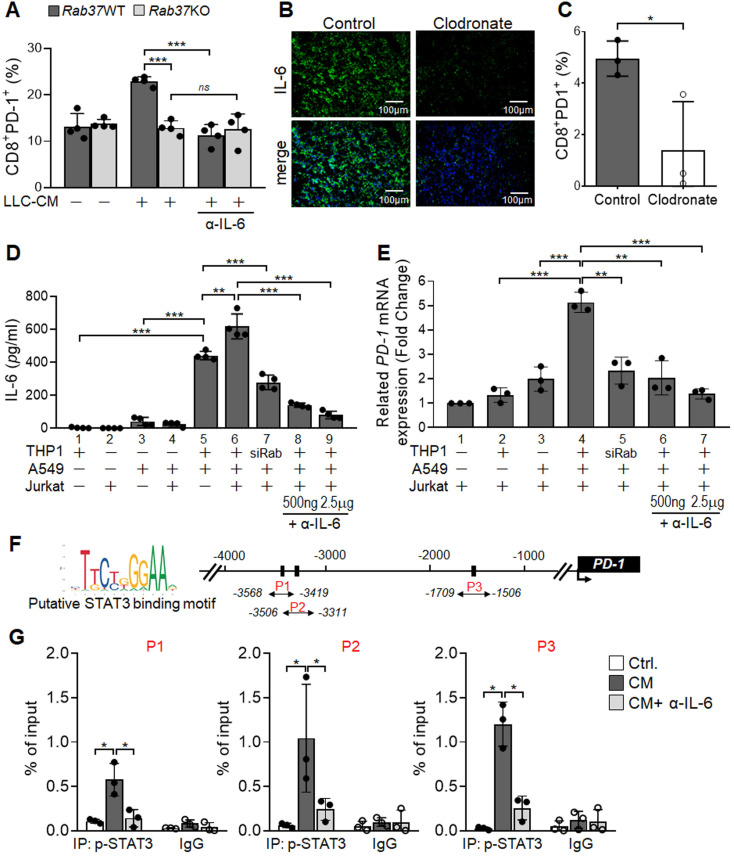
** Macrophage-derived IL-6 promotes PD-1 expression in T cells *via* the IL-6/STAT3 transcription axes*.* (A)** PD-1 expression on CD8^+^ T cells derived from* Rab37* WT and KO in the presence of α-IL-6 treatment. **(B, C)** Depletion of macrophages by clodronate-liposomes in mice reduced IL-6 level in LLC allografts (B) and decreased the PD-1 expression on the tumor-infiltrating CD8^+^ T cells (C). Scale bars: 100 µm. **(D-F)** Levels of IL-6 in the CM (D, E) and *PD-1* mRNA of Jurkat T cells (D, F) derived from of mono-culture of THP-1 macrophages, A549 cancer cells or Jurkat T cells or in those of co-cultured conditions as indicated. Levels of IL-6 (D, E) and *PD-1* mRNA (D, F) of Jurkat T cells co-cultured with THP-1 cells with Rab37 (siRab) knocked down or treated with α-IL-6 are shown. **(G)** ChIP-qPCR primers designed in P1, P2 and P3 regions of *PD-1* promoter are indicated below the map. Sequences of the STAT3 binding motif are shown (*left*) and STAT3 binding sites are specified as boxes in the map (top).** (H)** p-STAT3 bound to *PD-1* promoter. ChIP-qPCR assay was performed using anti-p-STAT3 antibody in Jurkat T cells treated with mixed CM with or without α-IL-6 treatment. IgG was used as negative control. Data represent mean ± SD. * p < 0.05; ** p < 0.01; *** p < 0.001; *ns* non-significant, Student's *t*-test.

Notably, blockade of IL-6 by α-IL-6 also attenuated PD-1 upregulation on WT CD8^+^ T cells upon LLC-CM treatment (Figure 4A). Therefore, we hypothesized that Rab37-mediated IL-6 secretion by macrophages promotes PD-1 expression on CD8^+^ T cells. Importantly, depletion of macrophages by clodronate liposomes injection into mice largely constrained IL-6 level in TME of LLC allografts (Figure 4B, S3A-B), supporting that macrophages are responsible for IL-6 production in TME *in vivo*. Strikingly, macrophage depletion not only elicited an immune activated TME to attenuate tumor growth (Figure S3C-F) but also reduced the tumor-infiltrating PD-1^+^CD8^+^ T cells (Figure 4C), suggesting that PD-1 expression on CD8^+^ T cells may be stimulated by the IL-6 produced by macrophages.

We undertook a co-culture approach to verify that Rab37-mediated IL-6 secretion in TME regulates PD-1 expression on T cells (Figure 4D). The cell types in the co-culture system included human THP-1 macrophages, human Jurkat T cells and A549 lung cancer cells. The A549 lung cancer cell line was chosen because its intrinsic Rab37 was expressed at a higher level than other lung cancer cells (Figure S4). The mono-cultured human THP-1 macrophages, human Jurkat T cells and A549 lung cancer cells or co-cultured A549 with Jurkat T cells secreted a little IL-6 into the CM (*Bars* 1-4, Figure 4E). IL-6 level significantly increased in CM from mixed culture of all three cell types (*Bar* 6, Figure 4E), and to a lesser extend in CM derived from co-culture of A549 cancer cells and THP-1 macrophages (*Bar* 5, Figure 4E). IL-6 level was decreased in co-culture of A549 cancer cells and THP-1 macrophages with Rab37 knocked down, suggesting that Rab37-mediated IL-6 secretion in macrophage cells plays an important role in cross-talk between cancer cells, macrophages and T cells (*Bar* 7, Figure 4E). Blockade of IL-6 by α-IL-6 attenuated the IL-6 levels in mixed CM in a dose-dependent manner (*Bars* 8-9, Figure 4E). These results reinforce the conclusion that macrophages largely contributed to the production of IL-6 in mixed culture of cancer cells, macrophages and T cells.

Next, we determined whether macrophage-derived IL-6 promoted PD-1 expression in T cells *via* the Rab37/IL-6/STAT3 transcription axis. To this end, we employed RT-qPCR analyses of *PD-1* mRNA expression in Jurkat T cells cultured under the co-culture conditions (Figure 4D). A significant increase of *PD-1* mRNA in Jurkat T cells cultured with mixed CM of cancer cells, macrophages and T cells was observed (*Bar* 4, Figure 4F), while culture with CM from Rab37 silenced THP-1 macrophages (*Bar* 5, Figure 4F) or blockade of IL-6 by α-IL-6 (*Bars* 6-7, Figure 4F) markedly attenuated* PD-1* expression in T cells, verifying the role of Rab37-mediated IL-6 derived from macrophages in mediating PD-1 expression in T cells.

To unravel the regulatory mechanisms of IL-6 secretion and PD-1 expression in T cells, we examined the binding of STAT3 transcription factor to the *PD-1* promoter in T cells. We first identified three STAT3 putative binding sites at *PD-1* promoter (Figure 4G). Next, chromatin-immunoprecipitation (ChIP)-qPCR results showed significant increase in p-STAT3 binding to the* PD-1* promoter and such binding was largely attenuated by α-IL-6 treatment at the three regions examined (Figure 4H). These results provide evidence that macrophage-derived IL-6, in the paracrine manner, promotes PD-1 membrane presentation on T cells *via* the Rab37/IL-6/STAT3 trafficking and transcription axes.

### CTLA-4 and IL-6 blockade combination therapy reduces tumor progression in a subcutaneous and an orthotopic model of lung cancer

Our results so far provide a new mechanism that Rab37 mediates the exocytosis of IL-6 to increase PD-1 expression on CD8^+^ T cells, suggesting that Rab37/IL-6/STAT3/PD-1 functions in the same axis in fostering an immunosuppressive lung TME. Indeed, the anti-tumor effects of anti-PD-1 antibody (α-PD-1) therapy were abrogated in *Rab37* KO mice (Figure S5A). In addition, the anti-tumor effects of JAK2/3 inhibitor AG490 was observed in WT mice but not in *Rab37* KO mice (Figure S5B), consistent with the hypothesis of Rab37/IL-6/STAT3/PD-1 functioning in the same axis.

**Figure 5:**
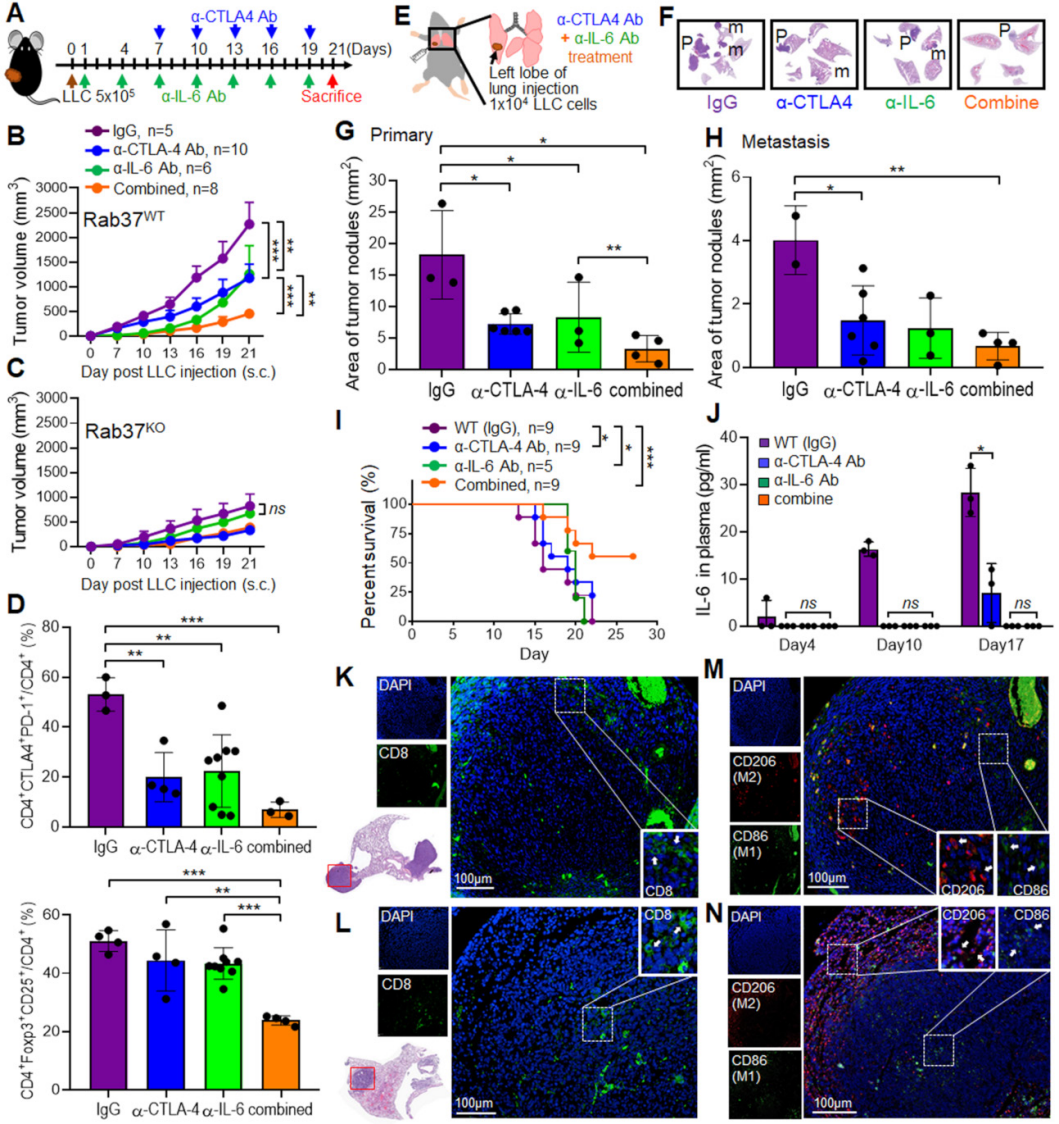
** Combined blockade of CTLA-4 and IL-6 provides synergistic anti-tumor efficacy with reactive immune responses* in vivo.* (A)** LLC (5 x 10^5^) subcutaneous model in *Rab37* WT and KO mice with α-IL-6 and/or α-CTLA-4 treatments. **(B, C)** Tumor volume of LLC allograft in *Rab37* WT (b) and KO (c) mice upon treatment. **(D)** Tumor-infiltrating PD-1^+^ and CTLA-4^+^ on CD4^+^ cells (*upper*) and Treg cells (*lower*). **(E)** Lung orthotopic model with LLC (1 x 10^4^ cells) injected into the left lung. The administration protocol of α-CTLA-4 and/or α-IL-6 was the same as that in subcutaneous model. **(F)** Representative H&E-stained sections of primary tumor (P) in the left lung and metastatic tumors (m) in the right lateral lungs in mouse of four treatment groups. **(G, H)** Overall area of tumor nodules of primary tumors (G) and metastatic tumors (H) in four treatment groups. **(I)** Survival curves were measured in LLC orthotopic mouse model.** (J)** The concentration of IL-6 in the plasma from treated mice measured on the indicated days.** (K, L)** Distribution of CD8^+^ T cells (*green*) by IF-IHC in orthotopic lung model in IgG control group (K) and combined treatment group (L). **(M, N)** Distribution of M1 (*green*) and M2 (*red*) macrophages in control group (M) and combined treatment group (N). Blue fluorescence represents the nucleus staining. Scale bar: 100 µm. Data represent mean ± SD. * p < 0.05; ** p < 0.01; *** p < 0.001; *ns* non-significant, Student's *t*-test.

In addition to PD-1, another inhibitory receptor CTLA-4 has been reported to inhibit T cell proliferation and activation 3, 4. The Rab37/IL-6/STAT3/PD-1 axis in immune cell models prompted us to determine whether concurrent blockade of IL-6 and CTLA-4 *in vivo* would provide a rational treatment to circumvent current PD-1-targeted immune-modulatory therapy for lung cancer. First, we tested the therapeutic efficacy of co-administration of α-IL-6 and anti-CTLA-4 antibody (α-CTLA-4) on LLC tumor growth in subcutaneous model (Figure 5A). We found that combination of α-IL-6 and α-CTLA-4 further inhibited tumor growth compared to IgG control groups and other single treatments (Figure 5B). Importantly, α-IL-6 treatment did not show any significant effect on *Rab37* KO mice, indicating that the beneficial effects of α-IL-6 and/or α-CTLA-4 treatment are dependent on the expression of Rab37 (Figure 5C). The FLOW results showed that the percentage of CTLA-4^+^PD-1^+^ on CD4^+^ cells (*Upper*, Figure 5D) and FoxoP3^+^CD25^+^ on CD4^+^ immunosuppressive Tregs (*Lower*, Figure 5D) were lower in tumor allografts from combined treatment compared to other single treatments. The reduction of CTLA-4^+^PD-1^+^CD8^+^ cells and Tregs could also be seen in the PBMC collected on day 10 during treatment (Figure S5C). Overall, α-IL-6 and α-CTLA-4 combination treatment reprogrammed to T helper 1 inflammation as determined by FLOW (Figure S5D).

Next, we tested the efficacy of co-administration of α-IL-6 and α-CTLA-4 on lung orthotopic model in which LLC cells were inoculated percutaneously into the left lateral thorax, and the tumor nodules in the right lungs of injected mice were considered as metastatic lesions (Figure 5E). Strikingly, combined treatment not only significantly decreased the primary tumor growth (Figure 5F-G) but also reduced lung to lung tumor metastases compared to the control (Figure 5F, H). Importantly, combined treatment prolonged mouse survival (Figure 5I). The ELISA results in animal model showed a positive correlation of plasma IL-6 level with tumor size (Figure 5J). Notably, treatment with antibody blocking IL-6, CTLA-4 or both did not result in any overt signs of toxicity measured by serum biochemistry analysis in all animals tested (Figure S5E). Therefore, α-IL-6 and α-CTLA-4 combination treatment led to effective and long-lasting anti-tumor activity.

### Combined blockade of IL-6 and CTLA-4 increases infiltration of CD8^+^ T cells and M1 macrophages into LLC tumors *in vivo*

Multi-color IF-IHC was performed on the LLC allograft tissues. There were less infiltrating CD8^+^ cells in tumor derived from IgG group and the CD8^+^ cells were mainly localized in tumor edge (Figure 5K). Combination treatment promoted more CD8^+^ cells infiltrating into tumor core (Figure 5L). Strikingly, more M2 macrophages (CD206) and less M1 (CD86) infiltrated into the tumor core in control IgG group (Figure 5M), whereas the infiltrating population of M1 macrophages increased in tumor core and M2 macrophages located toward the tumor boundaries after combined treatment (Figure 5N). These results demonstrated that combination of α-IL-6 and α-CTLA-4 therapies shaped TME to a T helper 1 immunity to suppress tumor growth and metastasis.

### Rab37 and IL-6 are concordantly expressed in infiltrating immune cells of tumor from NSCLC patients with poor prognosis

**Figure 6:**
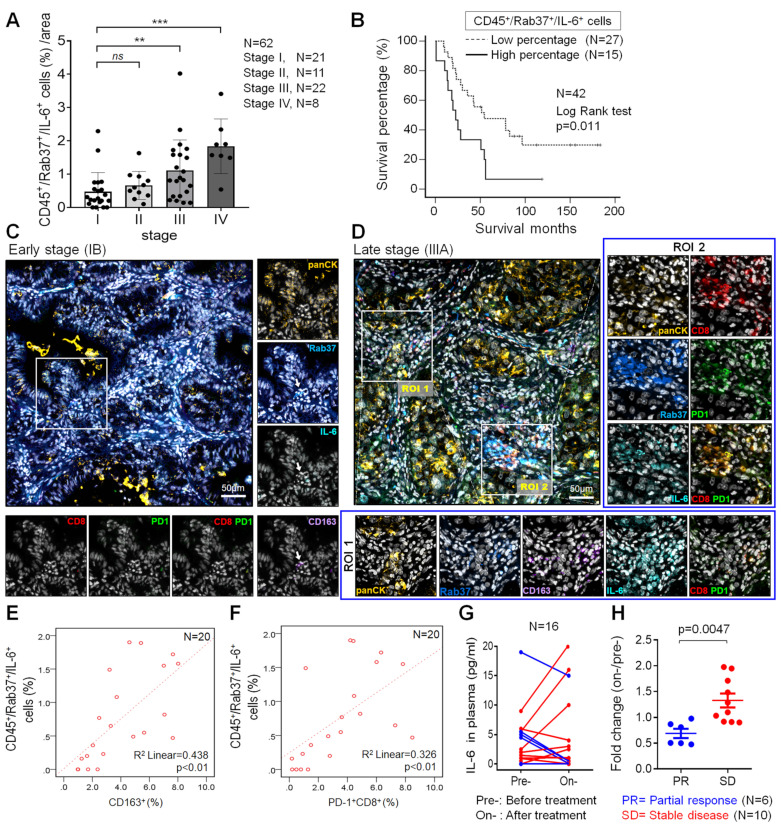
** Intratumoral CD45^+^Rab37^+^IL-6^+^ areas are enriched with M2-TAMs and** PD-1^+^CD8^+^** T cells in lung cancer patients. (A)** Proportion of infiltrated CD45^+^Rab37^+^IL-6^+^ cells correlated with the tumor progression stages (n = 62) (stage I *vs*. stage III, p < 0.01; stage I *vs*. stage IV, p < 0.001). **(B)** Overall survival curve of Kaplan-Meier method indicated that patients with highly intratumoral CD45^+^Rab37^+^IL-6^+^ cells had significantly poorer survival than patients with low infiltrated ones. *P* values determined using log-rank test. **(C, D)** Representative images showing that intratumoral Rab37^+^/IL-6^+^ cells coincide with a higher number of CD163^+^ M2-TAMs and CD8^+^PD-1^+^ T cells in the late stage patient (D, indicated by two regions of interest, ROIs) than that in the early one (C, indicated by an arrow). Corresponding ROIs were captured for individual analysis based on immunofluorescent staining for Rab37 (blue), IL-6 (cyan), CD8 (Red), PD-1 (green), CD163 (purple), panCK (yellow), and DAPI (gray). Scale bar: 50 µm. **(E, F)** Scatter plot showing the correlation between CD45^+^Rab37^+^IL-6^+^ cells with CD163^+^ cells (E) or with PD-1^+^CD8^+^ cells (F) in lung cancer patients. Pearson correlation coefficient, R square and *P*-value are shown. **(G, H)** Changes of IL-6 level in plasma from pretreatment to on-treatment of α-PD-1 blockade of lung cancer patients were measured (G). Fold changes of the IL-6 levels (on-/pre-IL-6) were analyzed (H). The lung cancer patients were grouped based on their clinical responses (PR: partial response, blue, n = 6; SD: stable disease, red, n = 10).

The relationship between stromal Rab37 and IL-6 expression has never been examined in TME of human cancer patients. Therefore, we performed multiplex IF-IHC with Rab37, IL-6 and CD45 staining for tumor-infiltrating immune cells on tumor specimens from 62 NSCLC patients. IF-IHC demonstrated that co-expression of intracellular Rab37 and IL-6 with tumor-infiltrating CD45^+^ immune cells (CD45^+^Rab37^+^IL-6^+^) was observed especially in late stage patients (Figure S6A-B). Patients with high proportion of CD45^+^Rab37^+^IL-6^+^ cells per region of interest (ROI) were significantly associated with advanced tumor stage (stages III and IV; *P* < 0.01) (Figure 6A).

We further investigated whether patients with intratumoral CD45^+^Rab37^+^IL-6^+^ expression profile correlated with cancer progression and poor prognosis. A total of 33.9% (21/62) patients showed CD45^+^Rab37^+^IL-6^+^ high expression (Table 1). Of note, patients with high intratumoral CD45^+^Rab37^+^IL-6^+^ expression were significantly associated with advanced tumor stage (*P* < 0.001), lymph node metastasis (*P* = 0.025) and distal metastasis (*P* = 0.003) (Table 1). Importantly, we performed Kaplan-Meier analysis on 42 NSCLC patients whose overall survival data were available. NSCLC patients with high proportion of intratumoral CD45^+^Rab37^+^IL-6^+^ cells correlated significantly with unfavorable outcome than those with low proportional ones (Figure 6B).

### Rab37^+^IL-6^+^ tumors are associated with intra-tumoral M2-TAMs (CD163^+^) and exhausted T cells (PD-1^+^CD8^+^) in lung cancer patients

To verify if CD45^+^Rab37^+^IL-6^+^ tumors elicited an immunosuppressive TME, we next examined whether the intratumoral M2-TAMs (CD163^+^) and exhausted T cells (PD-1^+^CD8^+^) were enriched in the area of CD45^+^Rab37^+^IL-6^+^ cells. Additional multiplex IF-IHC of CD163, CD8, PD-1 and panCK was performed on 20 tumor specimens, which were stained for Rab37, IL-6 and CD45. Two representative images are shown in Figure 6C-D. In the advanced stage patients whose tumors were highly enriched in CD45^+^Rab37^+^IL-6^+^, stromal Rab37 was accumulated around the CD163^+^ M2-TAMs (ROI 1) (*Lower*, Figure 6D) or PD-1^+^CD8^+^ exhausted T cells (ROI 2) (*Right*, Figure 6D). In contrast, early staged tumors displayed a low abundance of infiltrating M2-TAMs and PD-1^+^CD8^+^ exhausted T cells as well as scattered intratumoral IL-6, while Rab37 was mostly expressed in epithelial compartment (Rab37^+^panCK^+^) (Figure 6C). Quantitative analysis indicated that the regions enriched in CD45^+^Rab37^+^IL-6^+^ cells were accompanied by high infiltration of CD163^+^ (R square = 0.438, *P* < 0.01, n = 20) (Figure 6E) and/or PD-1^+^CD8^+^ cells (R square = 0.326, *P* < 0.01, n = 20) (Figure 6F). These clinical results demonstrated, for the first time, that stromal Rab37 was characterized by a distinct pro-tumor immune microenvironment with a high level of intratumoral IL-6 adjacent to M2-macrophages and PD-1^+^CD8^+^ exhausted T cells to elicit an immunosuppressive TME, leading to poor prognosis of NSCLC patients.

### Change in the plasma IL-6 level reflects the therapeutic efficacy of α-PD-1 treatment in lung cancer patients

Our ELISA results in animal model showed a positive correlation of plasma IL-6 level with tumor size (Figure 5J). In addition, our clinical data showed that IL-6^+^ signals in TME were stronger in tumor derived from advanced stage patients than those from early ones (Figure 6C). Therefore, we examined whether plasma level of IL-6 correlated with therapeutic efficacy of α-PD-1 treatment in NSCLC patients. ELISA assay was performed on plasma samples derived from 16 NSCLC patients receiving α-PD-1 immunotherapy whose sequential blood samples were available. Patients were divided into two groups: poor treatment response (SD, stable disease) and partial response (PR). Strikingly, SD patients were associated with greater increase in plasma IL-6 levels during α-PD-1 treatment (on-/pretreatment; median value, 1.38-fold) compared with PR patients (on-/pretreatment; median value, 0.72-fold) (Figure 6G-H). These results implicated that an increase in plasma IL-6 level during PD-1 blockade correlates with poor therapeutic responsiveness of NSCLC patients.

## Discussion

**Figure 7:**
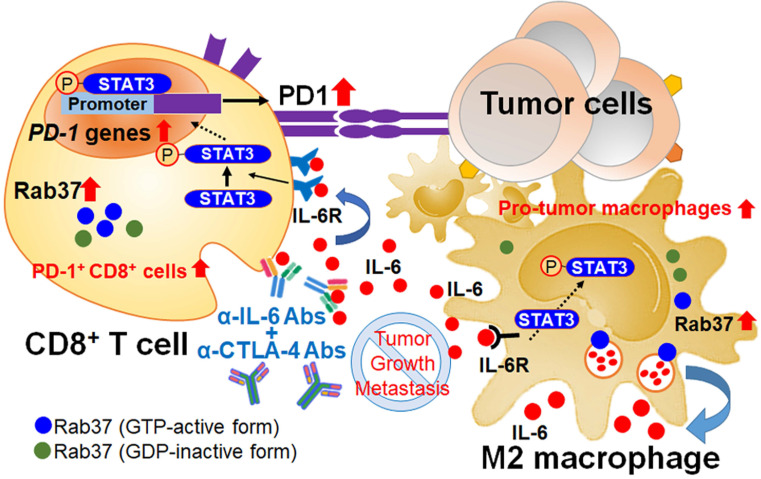
** Schematic diagram of Rab37/IL-6/STAT3/PD-1 trafficking and transcription axes elicits an immunosuppressive TME which is abrogated by IL-6 and CTLA-4 combined blockage in lung cancer.** Rab37-mediated IL-6 secretion acts in an autocrine manner in TAMs to promote STAT3 signal and pro-tumor M2 macrophage polarization. In addition, intratumoral IL-6 functions in a paracrine manner to up-regulate *PD-1* mRNA expression in CD8^+^ T cells through STAT3 transcriptional control and cause T cell exhaustion. Rab37/IL-6/STAT3/PD-1 functions in the same axis in fostering an immunosuppressive lung TME which can be converted to immuno-stimulatory environment with anti-tumor efficacy by combination treatment of α-IL-6 and α-CTLA-4 antibodies. Lung cancer patients with intratumoral Rab37^+^IL-6^+^ immune cells coinciding with CD163^+^ M2-TAM and PD-1^+^CD8^+^ exhausted T cells in their tumors show high lymph node metastasis and poor survival.

Here, we reveal novel findings that Rab37, a regulator of vesicle transport and protein trafficking, acts as a tumor promoter in stromal cells. Rab37 mediates IL-6 secretion and STAT3 transcription activation leading to M2 macrophage polarization and upregulation of PD-1 on CD8^+^ T cells, ultimately establishing an immunosuppressive TME (Figure 7). We and others have previously described the mechanism by which *Rab37* gene expression is downregulated by promoter hypermethylation in cancer cells 26, 27. Until now, promoter methylation of Rab37 has not been observed in immune cells where Rab37 is expressed in a pleiotropic manner. For example, Rab37 is expressed in secretory granules of mast cells to regulate mast cell degranulation 28. In cytotoxic CD8^+^ T cells, Rab37 has been found in recycling endosomes 29. In CD4^+^ T helper cells, Rab37 is suggested to be associated with secretion of IL-2, IL-4 and TNF-α 22. Interestingly, gene expression of Rab37 was found to be upregulated in skin wounds and LPS-stimulated macrophages indicating that Rab37 may participate in both tissue inflammation and repair 23. Together, these published results and our data suggest that Rab37 is a critical protein in modulating immune cells in physiological and pathological conditions.

IL-6 is a pleotropic pro-inflammatory cytokine, thereby contributing to the dysfunction of innate and adaptive immune responses against tumors 30. However, the secretion mode of IL-6 is not fully understood. In this study, we identified IL-6 as a secretory cargo protein of Rab37 in TAMs. A study has shown that TAM-derived IL-6 promotes mouse lung tumor growth through activation of STAT3 31. Notably, the downstream signal pathways of immunosuppressive cytokine transforming growth factor-β (TGF-β) can also promote T cell dysfunction in the TME. For example, Stephen and associates showed that tumor-derived TGF-β activates Smad3 to increase PD-1 expression by inhibiting the chromatin organizer Satb1 in tumor-reactive T cells 32. Furthermore, TGF-β combined with IL-6 induces the expression of transcription factor Maf, a potential driver of dysregulation of CD8^+^ T cells in TME 33. On the other hand, several previous studies showed that TLR4-induced NF-κB signaling pathway was able to facilitate IL-6 expression *via* stabilization of *IL-6* mRNA in macrophages 34, 35. Additionally, LLC tumor-induced increase of circulating TNF-α and IL-6 was abolished in TLR4 KO mice 36. These findings suggest that TLR4-mediated signaling may participate in IL-6 production, perhaps also for Rab37 activation, in lung M2-TAMs.

We discovered that macrophage-derived IL-6 served as a paracrine signal to up-regulate *PD-1* mRNA expression in T cells through STAT3 transcriptional control. Indeed, immunoreactivity of p-STAT3 in TME correlates with expression of PD-1^+^ stromal cell 37. Moreover, IL-6/STAT3 signal may cooperate with other factors in TME to induce *PD-1* mRNA expression in T cells 38. Previous studies reported that *Pdcd1* gene in T cells can be positively regulated by STAT3, NFATc1, FoxO1, and NF-κB, or negatively controlled by Blimp-1 and T-bet 39-42. Using ChIP assay, we identified three STAT3 binding sites at *Pdcd1* promoter at -3568 ~ -3419, -3506 ~ -3311 and -1709 ~ -1506, which are located close to the two previously reported DNase I hypersensitive sites at -3.7 kb and -1.1 kb 38. It is worth noting that these sites can also be bound by NFATc1 and NFκB 37. Moreover, chromatin landscape of *Pdcd1* gene locus was dynamically changed during T cell stimulation and cytokine exposure 38, 41, 43. Nevertheless, our model of the converged Rab37/IL-6/STAT3 trafficking and transcription axis adds a new layer of PD-1 transcriptional regulatory mechanisms.

Development of effective therapeutics to ameliorate the immunosuppressive TME in cancer patients is eagerly anticipated 44. Our results suggest that Rab37/IL-6/PD-1 functions in the same axis and thus provide a rationale design using combined treatment of α-IL-6 and α-CTLA-4 to promote anti-tumor efficiency*.* Several studies have shown that inhibition of IL-6 enhances the therapeutic efficacy of α-PD-L1 therapy in melanoma, colorectal cancer, pancreatic cancer and hepatocellular carcinoma 45-48. Intriguingly, the beneficial effect of combination therapy with α-IL-6 and α-PD-1 has not been reported, supporting our notion that IL-6 and PD-1 work in the same pathway. In addition, we found that plasma IL-6 level reflects the therapeutic efficacy of α-PD-1 treatment in NSCLC patients. Consistently, an increase in circulating IL-6 in anti-PD-1 therapy has been shown to correlate with the development of pathologic immune-related adverse events 49, 50. Therefore, our study not only provides an efficient way for patients with resistance to anti-PD1/PD-L1 therapy to recover from immunosuppressive status but also a potential biomarker using plasma IL-6 level for patients selection to receive dual IL-6/CTLA-4 blockade therapy.

Our clinical data corroborated with *in vitro* and *in vivo* experiments that infiltration of intratumoral Rab37^+^IL-6^+^ immune cells enriched with CD163^+^ M2 macrophages is a feature of high density of PD-1^+^CD8^+^ exhausted T cells. IL-6 is reported to induce the expression of M2-like macrophages and granulocytic myeloid-derived suppressor cells, and protumor T-regulatory/Th17 cell responses, whereas reducing anti-tumor ability of Th1 and CD8^+^ T cells 51, 52, 53. Note that increased level of IL-6 in patients treated with immune checkpoint blockade is associated with poor responses and survival 54-56. The intratumoral spatial pattern of Rab37^+^IL-6^+^CD163^+^CD8^+^PD1^+^ defined by IF-IHC or high IL-6 of plasma ELISA as biomarkers would identify patients in dire need of intensive surveillance and further combination therapy with α-IL-6 and α-CTLA-4 antibodies would improve their clinical outcome.

In conclusion, we found that Rab37-mediated exocytosis of IL-6 from macrophages promotes PD-1 upregulation in T cells *via* the Rab37/IL-6/STAT3 transcription axis to support lung cancer progression. Combined blockade of IL-6 and CTLA-4 suppressed lung tumor growth in mice by increasing tumor-infiltrating CD8^+^ T cells and M1 macrophages. Clinically, NSCLC patients with high intratumoral CD45^+^Rab37^+^IL-6^+^ or IL-6^+^CD163^+^CD8^+^PD-1^+^ immunoreactivity or an increase in plasma IL-6 level are found to be significantly associated with poor prognosis or therapeutic resistance. This study uncovers a novel function of Rab37 in suppressing tumor immunity and adds a new insight into the complexity of PD-1 regulation. Our findings provide a rationale and feasible combined therapeutic approach targeting IL-6 and CTLA-4 in lung cancer patients.

## Supplementary Material

Supplementary figures and tables.

Supplementary movie S1.

Supplementary movie S2.

Supplementary movie S3.

Supplementary movie S4.

Supplementary movie S5.

Supplementary movie S6.

## Figures and Tables

**Table 1:** Expression profile of intratumoral CD45^+^Rab37^+^IL6^+^ levels in relation to clinicopathological parameters in 62 NSCLC patients

Clinicopathologic parameters		CD45^+^Rab37^+^IL6^+^ cells (%)^a^		*p* value^b^
	Total	Low (%) (66.1%)	High (%) (33.9%)	
	N = 62	N = 41	N = 21	
**Stage**				
Stage I-II	32	28 (87.5)	4 (12.5)	
Stage III-IV	30	13 (43.3)	17 (56.7)	< 0.001
**N stage**^c^				
N0	27	22 (81.5)	5 (18.5)	
N ≥ 1	35	19 (54.3)	16 (45.7)	0.025
**M stage**^c^				
M0	53	39 (73.6)	14 (26.4)	
M ≥1	9	2 (22.2)	7 (77.8)	0.003

^a^ Categorical data were presented as the mean. ^b^ The data were analyzed by Pearson χ^2^ test. ^c^ N Stage: lymph node metastasis; M Stage: distal organ metastasis.
