# Supplementary material for: Converged Rab37/IL-6 trafficking and STAT3/PD-1 transcription axes elicit an immunosuppressive lung tumor microenvironment
Source: Theranostics. 2021 May 12;11(14):7029–44. doi: 10.7150/thno.60040 (PMC8171097; doi:10.7150/thno.60040)
Supplement: Supplementary file 1 — Supplementary figures and tables. [file thnov11p7029s1.pdf]

Converged Rab37/IL-6 trafficking and STAT3/PD-1 transcription axes elicit an immunosuppressive lung tumor microenvironment

I-Ying Kuo, You-En Yang, Pei-Shan Yang, Yu-Jou Tsai, Hong-Tai Tzeng, Hung-Chi Cheng, Wan-Ting Kuo, Wu-Chou Su, Chih-Peng Chang & Yi-Ching Wang

SUPPLEMENTAL MATERIALS AND METHODS

Bone-marrow-derived macrophages (BMDMs) isolation and culture conditions

Bone marrow cells were aseptically harvested from hind legs of 6-to-8 week-old wild-type (WT) or *Rab37* KO mice. All muscle tissues were removed and the femur and tibia were separated. Isolated bones were washed with completed DMEM containing 10 *n*g/mL macrophage colony-stimulating factor (M-CSF) (Peprotech, Cranbury, NJ, US). 3×10^6 isolated bone marrow cells were seeded in 10 cm dish with 10 ml complete DMEM containing 10 *n*g/mL M-CSF. BMDMs differentiated 7 days at 37 ^oC with 5% CO_2 in air and changed to fresh medium every 2 days during *ex vivo* culture.

$CD4^+$ and $CD8^+$ T cells isolation and stimulation

Splenocytes derived from WT and *Rab37* KO mice were obtained by mashing the spleen and removing erythrocytes using red blood cells lysis buffer. $CD4^+$ and $CD8^+$ T cells were isolated using anti-mouse CD4 magnetic particles and mouse CD8 T lymphocyte enrichment set (BD Bioscience, Franklin Lakes, NJ, US). Cells were cultured in medium containing 10% FBS (Gibco, Waltham, MA, US), 1% penicillin/streptomycin (Gibco), and 1% sodium pyruvate (Gibco) and incubated at 37 ^oC with 5% CO_2 in air. Purified naïve $CD4^+$ and $CD8^+$ T cells were stimulated using 2 μg/mL anti-CD3 together with 2 μg/mL anti-CD28 antibodies (BD Bioscience) for 24 h or 48 h.

Conditioned medium (CM) preparation, collection and treatment

LLC cells (5×10^5 cells in 10 cm dishes) were seeded and incubated overnight. On the second day, media were replaced with 5 ml fresh complete medium and cultured for 48 h. The CM were collected, filtered through sterile Millex® Filter Units (Merck Millipore, Burlington, Massachusetts, US) and stored at -80 ^{o}C. The cell viability of > 98% was monitored using trypan blue dye exclusion assay. In addition, CM were collected from supernatants of A549 and/or THP-1 cells seeded at a density of 5×10^5/mL to treat Jurkat cells (1×10^6). Jurkat cells were then harvested after 24 h for ELISA, RT-qPCR or ChIP analysis. In some experiments, α-IL-6 Ab was added to the CM at a concentration of 500 *n*g/mL or 2.5 μg/mL before being subjected to ELISA, immunoblotting or FLOW assay.

Enzyme-linked immunosorbent assay (ELISA)

The secretion levels of mouse IL-6 (DY406), mouse IFN-γ (DY485) and human IL-6 (DY206) levels were detected by a double antibody sandwich ELISA according to the manufacturer's instructions (R&D systems, Minneapolis, MN, US). Briefly, 96-well microplates were coated with 100 μl/well of the capture antibody (anti-mouse IL-6, 2.0 μg/mL; anti-human IL-6, 2.0 μg/mL; anti-IFN-γ, 4.0 μg/mL) overnight at room temperature. After blocking, 100 μl CM from cell lines or serum from patients was added and incubated overnight at 4 ℃. Subsequently, 100 μl/well of the detection antibody (anti-mouse IL-6, 75 *n*g/mL; anti-human IL-6, 50 *n*g/mL; anti-IFN-γ, 200*n*g/mL) was added and incubated for 2 h at room temperature. Finally, the reaction was read at an OD of 450 nm using an ELISA plate reader.

Measurement of mouse plasma cytokines by cytometric bead array

Peripheral blood samples were collected from mice to analyze the Th1/Th2/Th17 cytokines by the mouse Th1/Th2/Th17 set of cytometric bead array (BD Biosciences)

according to manufacturer's instructions. In brief, seven groups of beads with distinct fluorescence intensities have been coated with capture antibodies specific for IL-2, IL-4, IL-6, IFN-γ, TNF-α, IL-17A, and IL-10 proteins. The plasma samples or recombinant standards were mixed with these array beads, and then incubated with PE-bound detection antibodies. The concentration of each cytokine was determined by analyzing PE fluorescence intensity using CytoFLEX flow cytometer (Beckman Coulter, Duarte, CA, US).

Cell lines, plasmids, shRNAs, transfection and culture conditions

Human lung cancer cell line A549, THP-1 monocytes, and Jurkat T cells and mouse macrophage cell line RAW264.7, T cell line EL4, Lewis lung carcinoma (LLC) was purchased from the American Type Culture Collection. A549, LLC and RAW264.7 were maintained in DMEM (Gibco) while Jurkat, EL4 and THP1 were in RPMI (Gibco) with 10% Fetal Bovine Serum (FBS, Gibco) and 1% penicillin/streptomycin (Gibco), and cultured at 37 ^{o}C with 5% CO_2 in air.

cDNA of mouse *Rab37* was purchased from OriGene and PCR fragments amplified with primers containing designated mutation for $Rab37^{Q89L}$ and $Rab37^{T43N}$ were cloned into the pcDNA-His-V5 vector (Invitrogen, Waltham, Massachusetts, US). To generate RFP-tagged Rab37, cDNA of $Rab37^{WT}$, $Rab37^{Q89L}$ and $Rab37^{T43N}$ was cloned into pDsRed2-C1 vector (NovoPro Bioscience Inc., Shanghai, China). Plasmid pCMV3-IL-6-GFPspark was purchased from Sino Biological Inc. (Beijing, China). Plasmid transfection was carried out with IT-J reagent (Mirus Bio, Madison, Wisconsin, US) or lipofectamine (Invitrogen) according to manufacturer’s protocol. To knockdown mouse Rab37, cells were transduced with control and shRab37 lentiviral particles produced in packaging vector pCMV-△R8.91 envelop plasmid (pMD.G) and targeted shRNA pLKO.1 plasmid. The viruses were obtained from the RNAi core (Academia Sinica, Taipei, Taiwan). To knockdown human Rab37,

siGENOME SMARTpool siRNA against human Rab37 (#EntrezGene:326624) were purchased from Dharmacon (Lafayet, CO, US). These transfected or infected cells were harvested and applied to *in vitro* and *in vivo* studies. Plasmids used in these studies are listed in Table S1.

FLOW cytometry analysis

BMDMs were harvested, washed and re-suspended in staining buffer (2% FBS, 0.1% sodium azide in PBS) and then subjected to centrifugation of 400 *g* for 5 min. Staining buffer was removed and stained with F4/80, CD86 and CD206 (BD Biosciences) antibodies. Cells from spleen or tumor tissue were stained with anti-mouse CD8-APC, CD4-PerCP, CD25-PE-CF594, PD-1-BV421 and CTLA-4-PE (BD Bioscience) in addition to F4/80, CD86 and CD206. Tumor tissues were digested with 0.1 mg/mL collagenase (Sigma-Aldrich, St. Louis, Missouri, US) and 1mg/ml dispase II (Sigma-Aldrich) in serum-free DMEM for 30 min at 37 °C and meshed with complete medium. Resulting single-cell suspension were incubated on ice for 30 min, washed and fixed with 2% paraformaldehyde and kept in staining buffer. All antibodies were diluted to 1:200. After cell surface staining, intracellular Foxp3 was stained with Foxp3-BV421 (BD Bioscience) antibody, which was diluted to 1:200 with permeabilized buffer. The data were recorded by CytoFLEX (Beckman Coulter). The detailed antibodies conditions are listed in Table S2.

Total internal reflection fluorescence (TIRF) imaging

Cells were transfected with GFP-IL-6 for 48 h and 1 × 10^4 cells were re-seeded in 3.5 cm glass bottom dish before image analysis. TIRF microscopy system (Olympus IX81, Tokyo, Japan) equipped with a high sensitivity EMCCD Camera (iXOn3897, Andor technology, New York, NY, US) and a UPON 100X oil objective lens (NA = 1.49, Olympus) was used to capture 100-200 nm images below the plasma membrane

interface. To observe the Rab37-mediated trafficking events of GFP-IL-6, the images sequences were recorded in stream model every 3 s per frame for 1 min. For quantitation of vesicle trafficking event, we defined each green fluorescence spot as an IL-6-containing vesicle, and then tracked each vesicle trafficking distance with trackIT software (Olympus).

Quantitative reverse transcriptase-polymerase chain reaction (RT-qPCR) and chromatin immunoprecipitation assay (ChIP) assays

Total RNA was extracted using Trizol reagent (Invitrogen). Purified RNA was converted into cDNA by reverse transcription. Q-PCR was performed to analyze the mRNA expression of genes by using SYBR Green Master Mix (Invitrogen). For ChIP assay, Jurkat T cells (5×10^6) were cross-linked followed by preparation of nuclear lysates using Magna ChIP™ protein G Kit (Millipore). Nuclear lysates were sonicated to shear DNA to around 500 bp using QSONICA sonicator with cooling system (on 45 s, off 30 s, 40 cycles) followed by immunoprecipitation with 5 μg anti-p-STAT3(Y705) antibody (#9145, Cell Signaling, Danvers, MA, US). Q-PCR was carried out using ChIP products. Results were normalized with input control. The primer sequences used for RT-qPCR and ChIP analyses are listed in Table S3.

Multiplex fluorescence immunohistochemistry (IF-IHC)

IF-IHC was performed to detect the protein level of Rab37, IL-6 and PD-1 and localization of $CD8^+$ T cells, Tregs, $CD86^+$, $CD206^+$, $CD45^+$ or $CD163^+$ cells in allografts from WT and *Rab37* KO mice or tumor specimens from lung cancer patients. The process was performed according to the manufacturer's instructions (Opal stain kit #NEL810001KT, Akoya Biosciences, Marlborough, MA, US). After antigen retrieval and blocking, slides were incubated with primary antibody. The next day, slides were incubated with polymer HRP Ms+Rb for 10 min and Opal

fluorophore for 10 min at room temperature. For staining of another primary antibody, antigen retrieval steps were repeated. DAPI was then applied for nucleus staining. The images were captured using Olympus FV3000 confocal microscope with FV31S-SW software (Olympus) to analyze the immunoractivity and co-localization signals. The antibodies and the experimental conditions were listed in Table S2.

Lymphocyte-mediated cytotoxicity against cancer cell assay

Splenocytes freshly isolated from WT and *Rab37* KO mice were stimulated using 2 μg/mL anti-CD3 together with 2 μg/mL anti-CD28 antibodies (BD Bioscience) for 24 h. These WT and *Rab37* KO mouse effecter splenocytes (E) were then co-cultured with luciferase-transduced mouse LLC cancer target cells (T) at 1:1, 2:1, 5:1, 10:1 in 96-well. Lymphocyte-mediated cytotoxicity against cancer cells was detected by loss of luciferase-transduced mouse LLC cancer after 48h co-culture by Dual-Luciferase® Reporter Assay System kit (#E1910, Promega, Madison, Wisconsin, US) using the protocols provided by manufacturer.

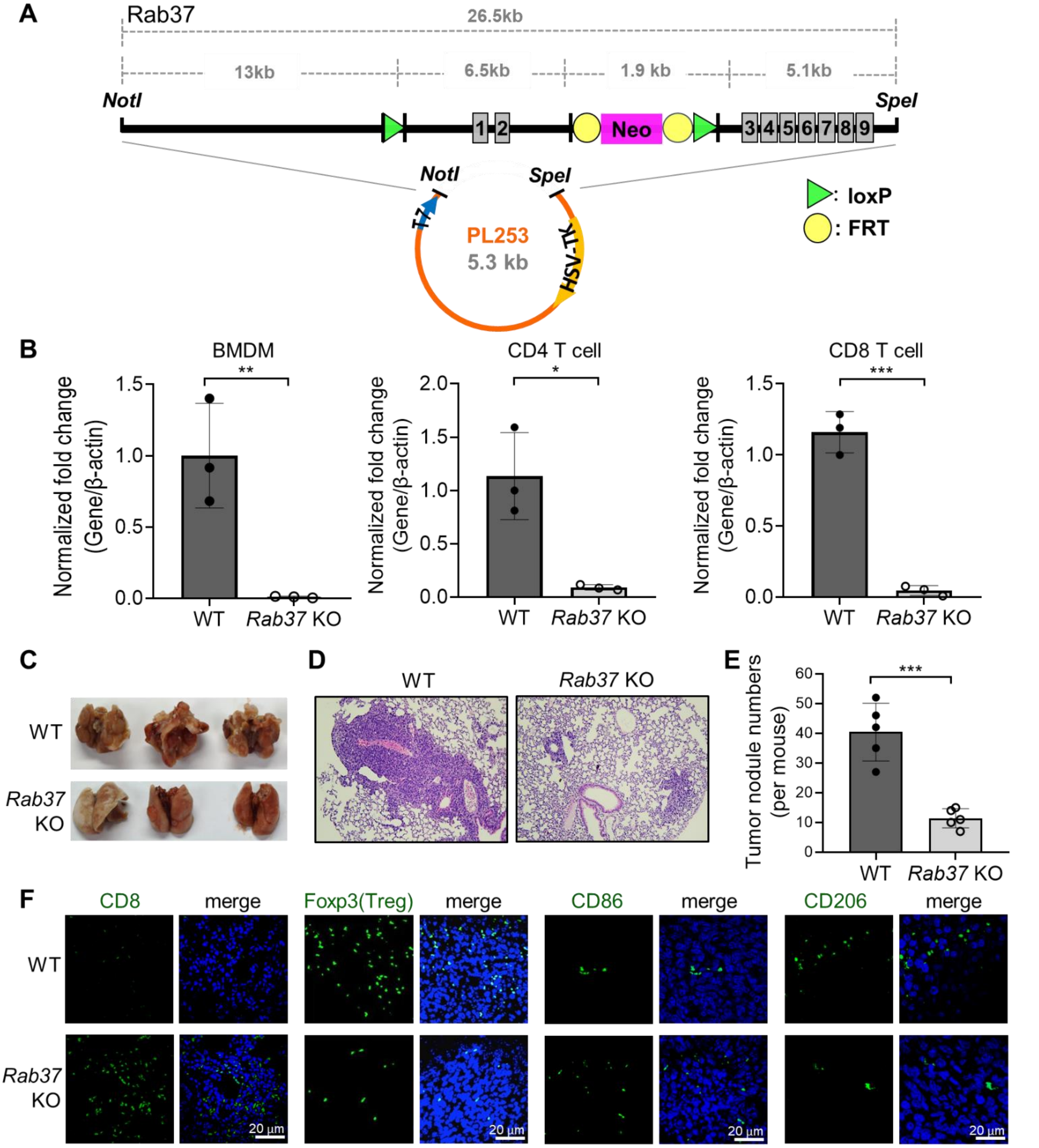

Figure S1. Rab37 in stromal cells fosters an immunosuppressive TME to promote lung cancer metastases *in vivo*. (A) Map of the PL253 vector containing *LoxP* flanked *Rab37* gene for Rab37-knockout. The *Rab37* gene was deleted by using *Cre-LoxP* system in C57BL/6J mice to establish the whole body deficiency of *Rab37* mice. **(B)** RT-qPCR confirmed *Rab37* mRNA deficiency in BMDMs, $CD4^+$ and $CD8^+$ T cells derived from *Rab37* KO mice. **(C-E)** Representative lung images (C) as well as H&E stains (D) and quantification of metastatic lung nodules (E) in *Rab37* WT or KO mice by tail-vein injection of LLC (1×10^5 cells) metastasis model. The size and number of metastatic cancer nodules in lungs were lower in *Rab37* KO mice than in WT mice. **(F)** Tumor-infiltrating T cells and macrophages of lung collected on day 21 were analyzed by IF-IHC. Scale bars: 20 μm. Data represent mean ± SD. * $p < 0.05$; ** $p < 0.01$; *** $p < 0.001$, Student's *t*-test.

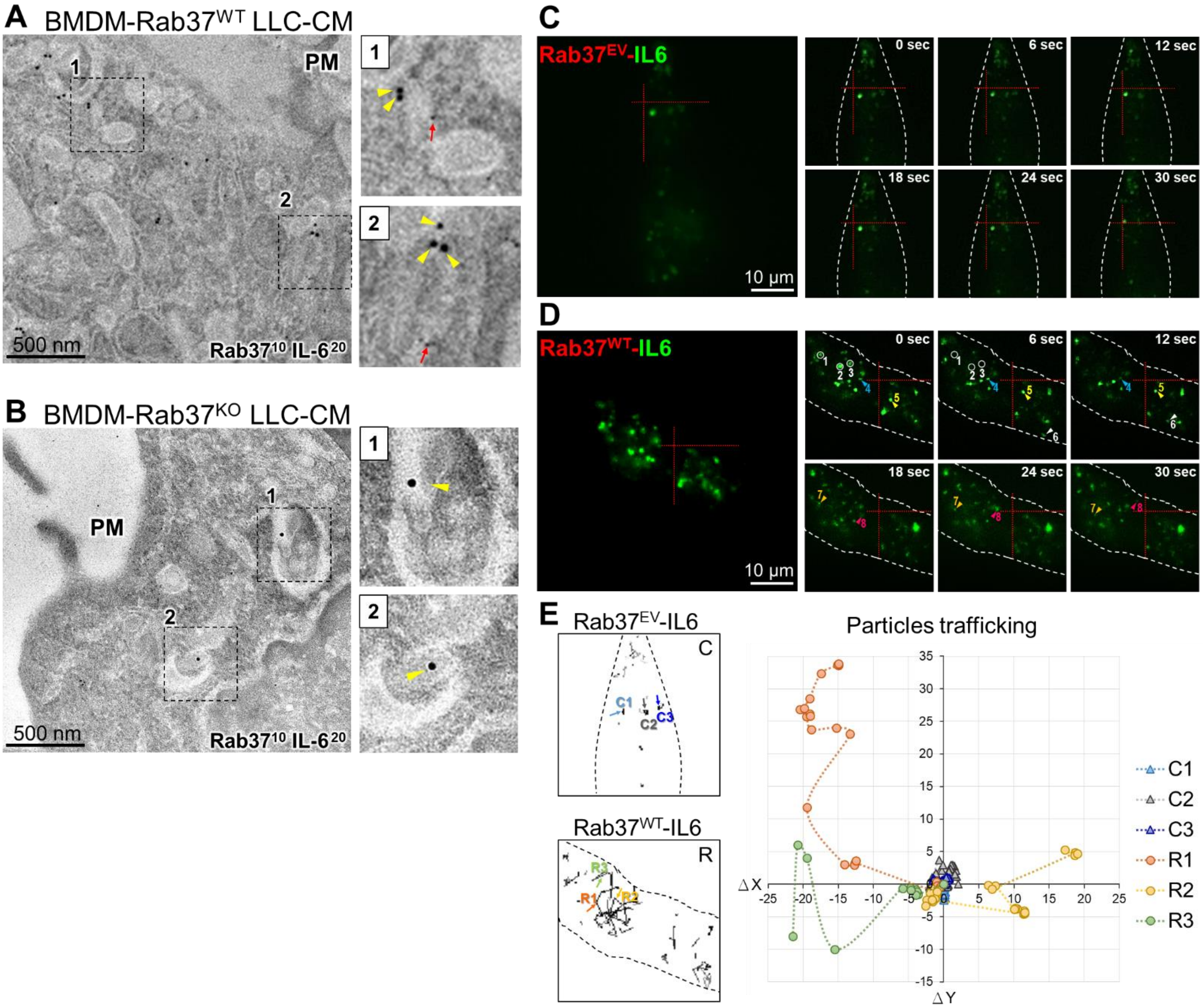

Figure S2. Ultrastructure of co-localized Rab37 and IL-6 in BMDMs and Rab37/IL-6 trafficking dynamic in macrophages. (A, B) Ultrastructural localization of Rab37 (10 nm of gold, red arrow) and IL-6 (20 nm of gold, yellow triangle) illustrated by immuno-EM images of *Rab37* WT BMDM (A) or *Rab37* KO BMDM (B) treated with LLC-CM. PM: plasma membrane. Scale bars: 500 nm. Enlarged images shown in insets of the representative regions. **(C, D)** Selected frames from TIRF movies of EV (C) or Rab37^{WT} (D) RAW264.7 cells co-transfected with GFP-tagged IL-6. Enlarged images of the indicated areas with time intervals in seconds are shown (*right*). Circle and arrow indicate vertical and horizontal trafficking vesicle, respectively. Scale bars: 10 μm. **(E)** For quantification of vesicle trafficking event, we defined three vesicles for EV group (C1, C2 and C3) and Rab37^{WT} (R1, R2 and R3), and then tracked each particle trafficking distance with trackIT software.

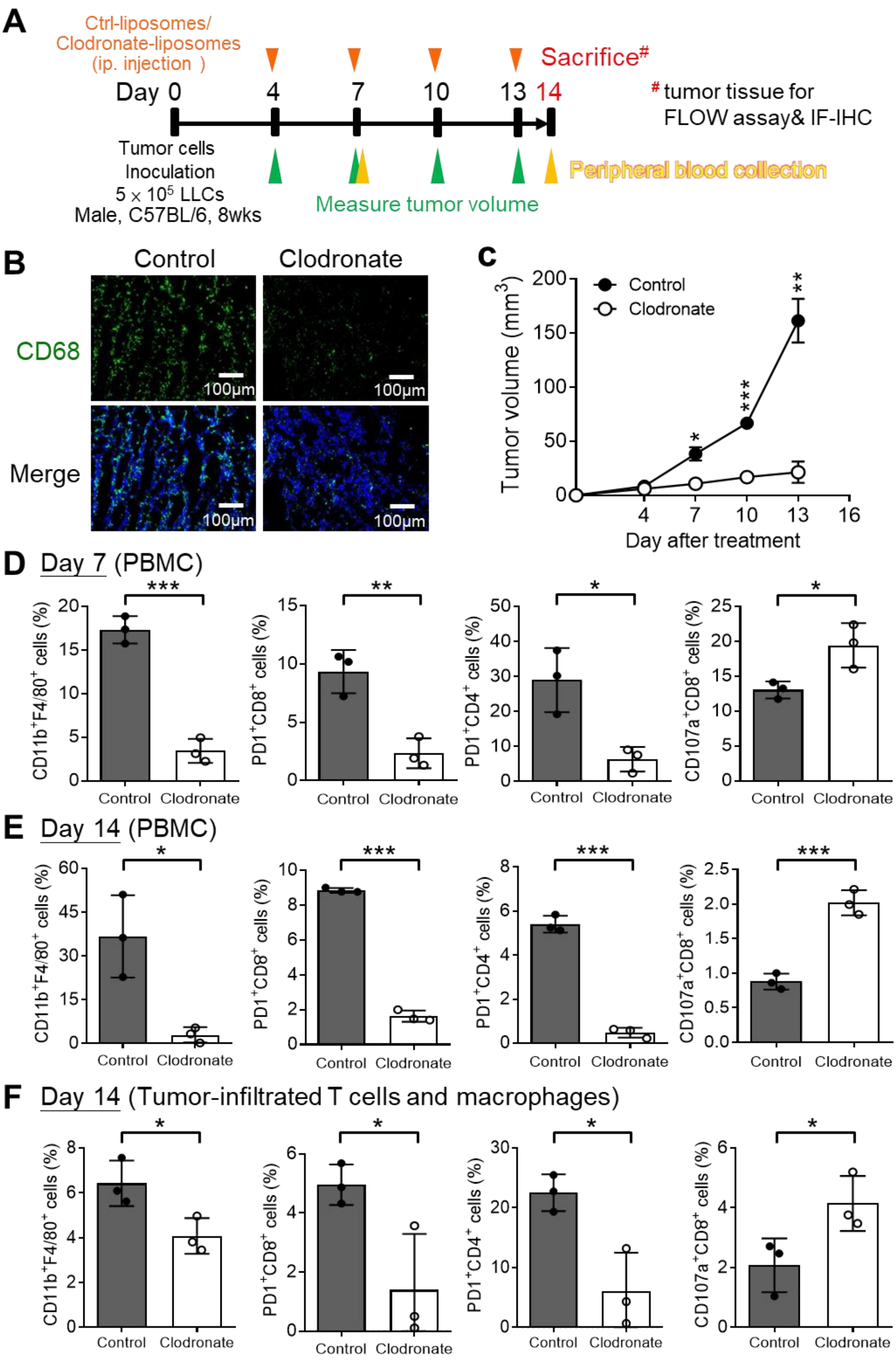

Figure S3. Macrophage depletion by clodronate-liposomes in mice reduces IL-6 level in LLC allograft which characterizes an immune active TME. (A) Treatment protocol of clodronate-liposomes and assay time points in mice. **(B)** IF images of CD68 macrophage marker in end point LLC tumor derived from control and clodronate-liposomes treated mice. Scale bars: 100 μm. **(C)** Tumor growth of LLC in control and clodronate-liposomes treated mice. **(D, E)** FLOW analysis of surface markers on circulating macrophages of peripheral blood mononuclear cell (PBMC) samples collected on day 7 (D) or day 14 (E). **(F)** FLOW analyses of tumor-infiltrating macrophages, $CD4^+$ and $CD8^+$ T cells in endpoint LLC allografts derived from control and clodronate-liposomes treated mice. Data represent mean ± SD. *p < 0.05; ** p < 0.01; *** p < 0.001, Student's *t*-test.

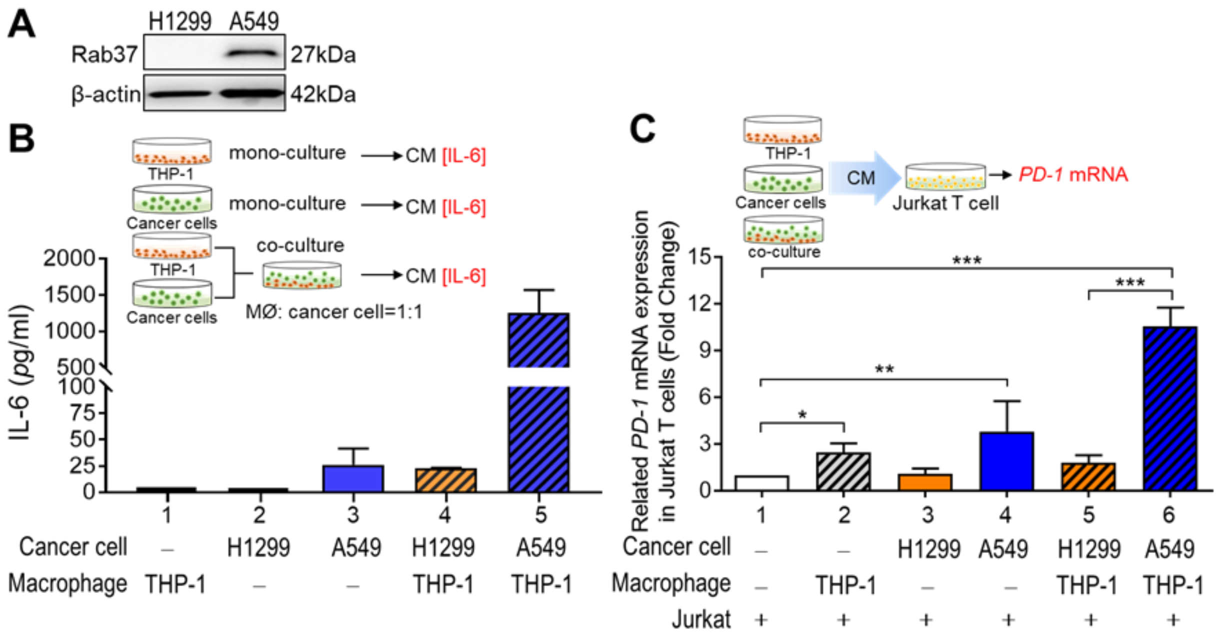

Figure S4. Rab37 mediates IL-6 secretion to regulate *PD-1* mRNA expression in T cells. (A) Basal level of Rab37 protein in two human lung cancer cells H1299 and A549. **(B)** Level of IL-6 in the CM and **(C)** *PD-1* mRNA of Jurkat T cells under the culture conditions as indicated. Data represent mean ± SD. *p < 0.05; ** p < 0.01; *** p < 0.001, Student's *t*-test.

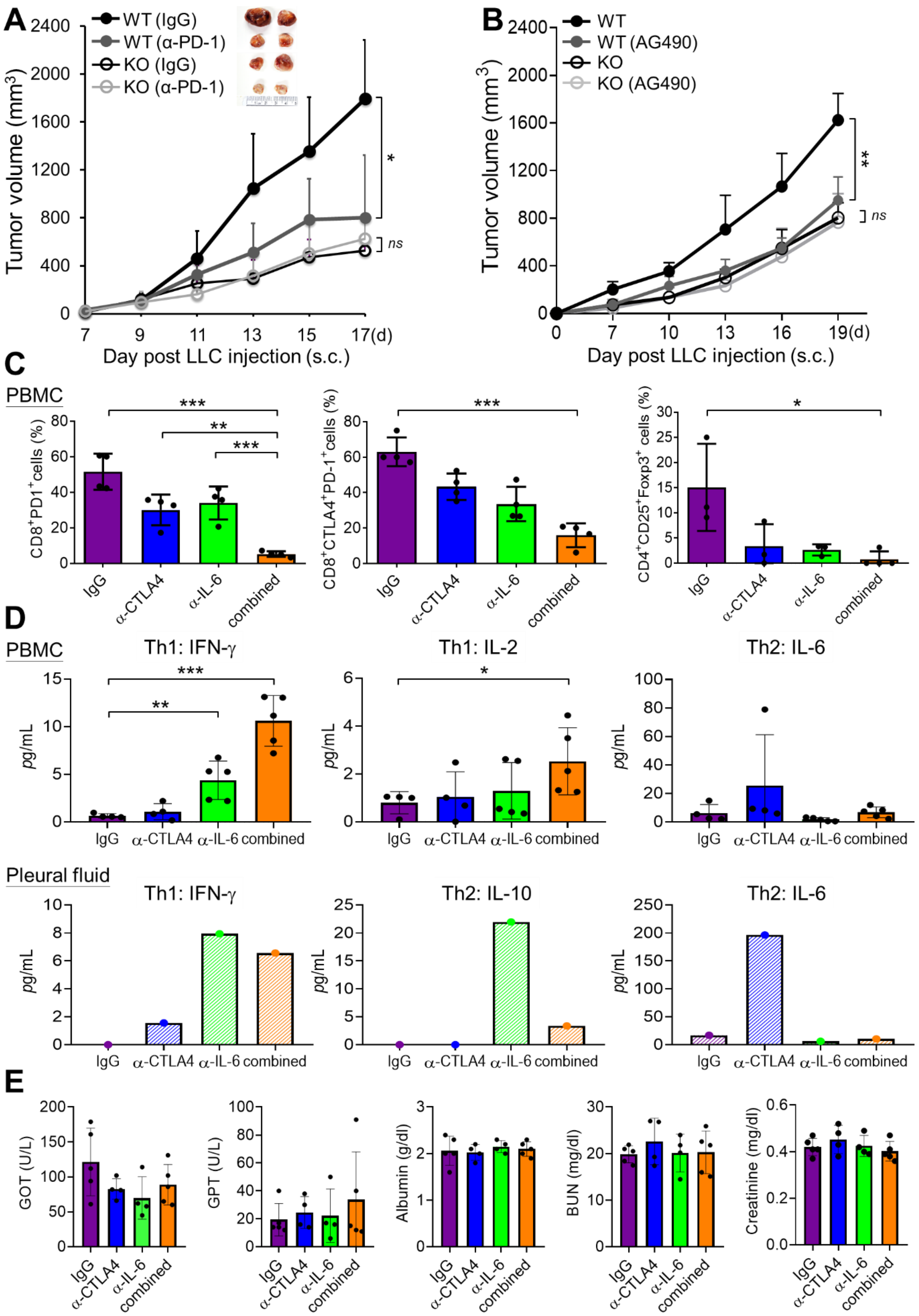

Figure S5. Combined blockade of CTLA-4 and IL-6 reactivates immune responses and shows no serum toxicity *in vivo*. (A, B) *In vivo* evidence showing that Rab37/IL-6/STAT3/PD-1 functions in the same axis. LLC (5 x 10^5) subcutaneous model in *Rab37* WT and KO mice treated with α-PD-1 antibody treatments (A) or STAT3 inhibitor AG490 (b). **(C)** FLOW analysis results of PD-1^+ and CTLA-4^+ CD8^+ cells and Treg cells in PBMC at the end point of four treatment groups. **(D)** α-IL-6 and α-CTLA-4 combination

treatment reprogrammed to T helper 1 (Th1) inflammation measured by FLOW assay of Th1/Th2 markers in PBMC (*upper*) and pleural fluid (*lower*). **(E)** Serum biochemical examinations of treated mice revealed no significant adverse effects upon combination treatment with α-IL-6 and α-CTLA-4. Data represent mean ± SD. * $p < 0.05$; ** $p < 0.01$; *** $p < 0.001$; *ns* non-significant, Student's *t*-test.

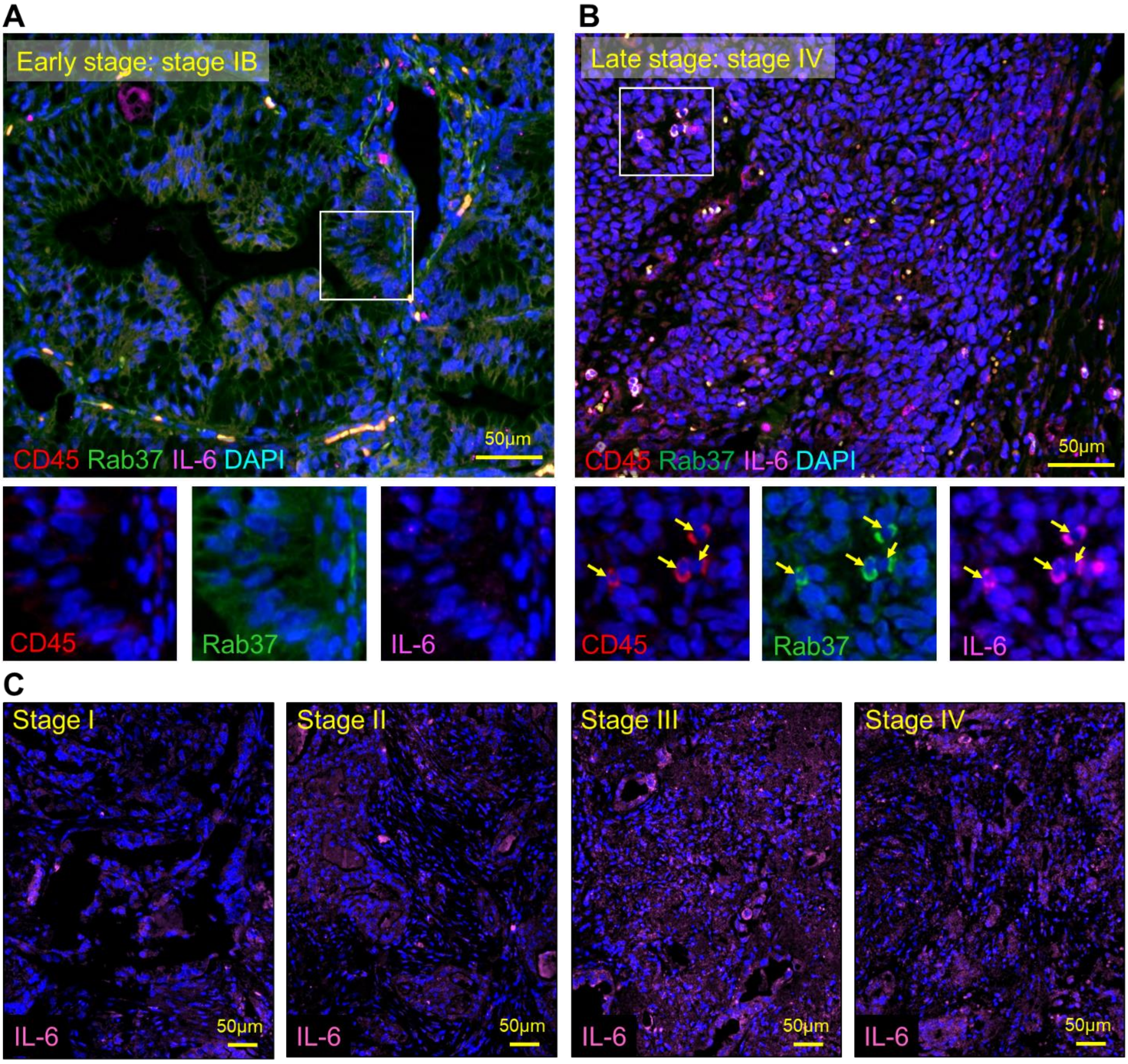

Figure S6. Intratumoral CD45$^+$Rab37$^+$IL-6$^+$ expression profile correlates with cancer progression. (A, B) Representative images of CD45$^+$Rab37$^+$IL-6$^+$ immune cells showing increased infiltration into the tumor core in the advanced stage patient (B), while CD45$^+$Rab37$^+$IL-6$^+$ immune cells at the tumor margins in the early one (A). CD45: red, Rab37: green, IL-6: magenta, Scale bar: 50 μm. **(C)** Confocal IF-IHC images showed that the expression levels of IL-6 in human lung cancer tissues significantly correlated with the tumor progression stages.

Movie S1. Time-lapse movie of confocal images in empty vector (EV) RAW264.7 cells expressing GFP-tagged EV and RFP-tagged IL-6. Images were captured with confocal microscope at 488 and 561 nm laser every 1 s over a period of 1 min. Time intervals in minutes and seconds are shown. Stills corresponding to frames from 00:04 to 00:34 of this movie are presented in Figure 2I (EV). Scale bars: 10 μm.

Movie S2. Time-lapse movie of confocal images in Rab37 wild-type ($Rab37^{WT}$) RAW264.7 cells expressing GFP-tagged $Rab37^{WT}$ and RFP-tagged IL-6. Images were captured with confocal microscope at 488 and 561 nm laser every 1 s over a period of 1 min. Time intervals in minutes and seconds are shown. Stills corresponding to frames from 00:08 to 00:38 of this movie are presented in Figure 2J ($Rab37^{WT}$). Scale bars: 10 μm.

Movie S3. Time-lapse movie of confocal images in Rab37 active Q89L ($Rab37^{Q89L}$) RAW264.7 cells expressing GFP-tagged $Rab37^{Q89L}$ and RFP-tagged IL-6. Images were captured with confocal microscope at 488 and 561 nm laser every 1 s over a period of 1 min. Time intervals in minutes and seconds are shown. Stills corresponding to frames from 00:00 to 00:30 of this movie are presented in Figure 2K ($Rab37^{Q89L}$). Scale bars: 10 μm.

Movie S4. Time-lapse movie of confocal images in Rab37 inactive T43N ($Rab37^{T43N}$) RAW264.7 cells expressing GFP-tagged $Rab37^{T43N}$ and RFP-tagged IL-6. Images were captured with confocal microscope at 488 and 561 nm laser every 1 s over a period of 1 min. Time intervals in minutes and seconds are shown. Stills corresponding to frames from 00:00 to 00:30 of this movie are presented in Figure 2L ($Rab37^{T43N}$). Scale bars: 10 μm.

Movie S5. Time-lapse movie of TIRF images in empty vector (EV) RAW264.7 cells expressing EV and GFP-tagged IL-6. Images were captured with TIRF microscope at 491 and 561 nm laser every 3 s over a period of 46 s. Time intervals in minutes and seconds are shown. Stills corresponding to frames from 00:16 to 00:46 of this movie are presented in Figure S2C ($Rab37^{EV}$-IL6). Scale bars: 20 μm.

Movie S6. Time-lapse movie of TIRF images in Rab37 wild-type ($Rab37^{WT}$) RAW264.7 cells expressing $Rab37^{WT}$ and GFP-tagged IL-6. Images were captured with TIRF microscope at 491 and 561 nm laser every 3 s over a period of 46 s. Time intervals in minutes and seconds are shown. Stills corresponding to frames from 00:03 to 00:33 of this movie are presented in Figure S2D ($Rab37^{WT}$-IL6). Scale bars: 20 μm.

Table S1. The plasmids and their characteristics used in the current study.

Plasmid	Target	Insert (bp)	Function	Source
pcDNA3.1-V5/His-vector	None	--[a]	Vector control	Invitrogen
pcDNA3.1-V5/His-mRab37^{WT}	Mouse Rab37^{WT}	672	Overexpression	This work[b]
pcDNA3.1-V5/His-mRab37^{Q89L}	Mouse Rab37^{Q89L}	672	Overexpression	This work[b]
pcDNA3.1-V5/His-mRab37^{T43N}	Mouse Rab37^{T43N}	672	Overexpression	This work[b]
pDsRed2-C1-vector	None	-[a]	Vector control	NovoPro Bioscience Inc.
pDsRed2-C1-hRab37^{WT}	Human Rab37^{WT}	672	TIRF	This work[c]
pLenti-C-mGFP	None	–[a]	Vector control	OriGene
pLenti-C-mRab37^{WT}-mGFP	Mouse Rab37^{WT}	672	Real-time confocal	OriGene
pLenti-C-mRab37^{TN}-mGFP	Mouse Rab37^{TN}	672	Real-time confocal	This work[d]
pCMV3-C-GFPSpark Control Vector	None	--[a]	Vector control	Sino Biological Inc.
pCMV3-hIL-6-GFPSpark	Wild type human IL-6	639	TIRF	Sino Biological Inc.
pCMV3-mIL-6-OFPSpark	Wild type mouse IL-6	636	Real-time confocal	Sino Biological Inc.
shRNA-mRab37-pLKO.1	Mouse Rab37WT	--[e]	Knockdown	Academia Sinica

[a] The plasmid is used as a backbone vector therefore there is no inserted DNA fragment.

[b] Rab37-WT, Q89L or T43N was PCR-amplified with designated mutation at the primer sequences and cloned into pcDNA3.1-V5/His expression vector to generate V5/His-tagged Rab37 expression vector.

[c] Mouse Rab37 cDNA was PCR-amplified and cloned into pDsRed2-C1 expression vector to generate RFP-tagged Rab37 expression vector.

[d] Mouse Rab37-T43N was PCR-amplified with designated mutation at the primer sequences and cloned into pLenti-C-mGFP expression vector to generate GFP-tagged Rab37 expression vector.

[e] shRab37 lentiviral particles were produced in packaging vector pCMV-△R8.91 envelop plasmid (pMD.G) and targeted shRNA pLKO.1 plasmid. The viruses were obtained from the RNAi core (Academia Sinica, Taipei, Taiwan).

Table S2. Antibodies and their reaction conditions used in the current study.

Target	KD	Raised in	Application	Dilution	Source	Catalog no.
Rab37	27	Rabbit	Western blot	1:1000	Proteintech	13051-1-AP
			Immunofluorescence	1:2000		
			Immunofluorescence-IHC	1:5000		
IL-6	24	Rabbit	Western blot	1:250-1:500[b]	Genetex	GTX110527
			Immunofluorescence	1:2000		
			Immunofluorescence-IHC	1:1000		
PD-1	32	Rabbit	Western blot	1:1000	Genetex	GTX31309
			Immunofluorescence-IHC	1:2000		
			Immunofluorescence	1:2000	Genetex	GTX128435
	--[a]	Mouse	Flow cytometry (APC)	1:200	BD Bioscience	562671
		Human	Flow cytometry (BB515)	1:200	BD Bioscience	564494
TIM-3	--[a]	Mouse	Flow cytometry (BV421)	1:200	BD Bioscience	747626
p-STAT-3 (Y705)	88	Rabbit	Western blot	1:500	Abcam	Ab76315
			Immunofluorescence	1:2000		
	--[a]		ChIP	1:100	Cell signaling	#9145
STAT-3α/β	86/91	Mouse	Western blot	1:500	Santa Cruz	Sc-8019
V5 tag	--[a]	Mouse	Western blot	1:5000	Invitrogen	46-0705
			Immunoprecipitation	1:150		
β-actin	42	Mouse	Western blot	1:5000	Genetex	GTX26276
GAPDH	37	Mouse	Western blot	1:1000	Santa Cruz	Sc-32233

CD45	--[a]	Rabbit	Immunofluorescence-IHC	1:1000	Abcam	ab40763
CD8	--[a]	Mouse	Flow cytometry (FITC)	1:200	BD Bioscience	553031
		Rabbit	Immunofluorescence-IHC	1:3000	Abcam	ab217344
CD4	--[a]	Mouse	Flow cytometry (PerCP)	1:200	BD Bioscience	561090
F4/80	--[a]	Mouse	Flow cytometry (PE)	1:200	BD Bioscience	565410
CD68	--[a]	Mouse	Immunofluorescence-IHC	1:100	Invitrogen	14-0681-82
CD68	--	Mouse	Immunofluorescence-IHC	1:2000	Abcam	ab233172
CD163	--[a]	Mouse	Immunofluorescence-IHC	1:100	Leica	NCL-L-CD163
panCK	--[a]	Mouse	Immunofluorescence-IHC	1:3000	Abcam	ab86734
CD11b	--[a]	Mouse	Flow cytometry (BB515)	1:200	BD Bioscience	564454
CD86	--[a]	Mouse	Immunofluorescence-IHC	1:1000	Genetex	GTX34569
		Mouse	Flow cytometry (BB605)	1:200	BD Bioscience	563055
CD206	--[a]	Rabbit	Immunofluorescence-IHC	1:1000	Abcam	ab64693
		Mouse	Flow cytometry (Alexa647)	1:200	BD Bioscience	565250
CD25	--[a]	Mouse	Flow cytometry (PE)CF594)	1:200	BD Bioscience	562695
Foxp3	--[a]	Mouse	Flow cytometry (BV421)	1:200	BD Bioscience	562996
CTLA-4	--[a]	Mouse	Flow cytometry (PE)	1:200	BD Bioscience	561718
CD107a	--[a]	Mouse	Flow cytometry (PerCP)	1:200	BioLegend	121626
DAPI	--[a]	-	Immunofluorescence	-[c]	Genetex	GTX30920

[a] Molecular weight is not applicable to this antibody in such an application.

[b] 1:250 is for vesicle isolation blotting; 1:500 is for total cell lysate blotting.

[c] DAPI is a commercial product for nuclear staining.

Table S3. The primers used in the current study.

Gene	Primer	Sequences (5'→ 3')	Application[a]	PCR size (bp)	Tm (°C)
Mouse *Actin* mRNA	Forward	GGT CCA CAC CCG CCA CCA G	RT-qPCR	75	60
	Reverse	CAC ATG CCG GAG CCG TTG TC			
Mouse *Rab37* mRNA	Forward	AAC TAC GAT CTC ACC GGC AA	RT-qPCR	248	60
	Reverse	AGC AAA GCC TGA GCA TCT CG			
Human *Actin* mRNA	Forward	GGC GGC ACC ACC ATG TAC CCT	RT-qPCR	202	60
	Reverse	AGG GGC CGG ACT CGT CAT ACT			
Human *PDCD1*[b] mRNA	Forward	GGC CAG GAT GGT TCT TAG ACT	RT-qPCR	147	60
	Reverse	GGT ACC AGT TTA GCA CGA AGC T			
Human *PDCD1*[b] promoter	Forward	AAT GCC CTG GAT TGA ATC TG	ChIP-P1-qPCR	150	60
	Reverse	CTC CCC CAT CTC CTT TTC TC			
	Forward	CCT CTG TGA GGA AGG AGC AG	ChIP-P2-qPCR	196	60
	Reverse	CCC AGT AGG CTT CCA CTG TC			
	Forward	TTG GTG GTG AGG AGA CCT TC	ChIP-P3-qPCR	204	60
	Reverse	GAG CCT TGT TCC AAC CTC TG			
Arg1 mRNA	Forward	CCA CAG TCT GGC AGT TGG AAG	RT-qPCR	106	60
	Reverse	GGT TGT CAG GGG AGT GTT GAT G			
Fizz1 mRNA	Forward	CCT GCT GGG ATG ACT GCT A	RT-qPCR	157	60
	Reverse	TGG GTT CTC CAC CTC TTC AT			

Ym1 mRNA	Forward	GCC ACT GAG GTC TGG GAT GC	RT-qPCR	113	60
	Reverse	TCC TTG AGC CAC TGA GCC TTC			
NOS2 mRNA	Forward	TGA CGC TCG GAA CTG TAG CAC	RT-qPCR	98	60
	Reverse	TGA TGG CCG ACC TGA TGT T			
IL-12 mRNA	Forward	GCG GAG CTG CTA CAC TCT CT	RT-qPCR	125	60
	Reverse	GAC CTG AAC GCA GAA TGT CA			

[a] RT-qPCR: Quantitative reverse-transcriptase polymerase chain reaction; ChIP: chromatin immunoprecipitation polymerase chain reaction.

[b] *PDCD1*: *Programmed Cell Death 1*, Gene codes for PD-1.

Table S4. Characteristics of the NSCLC patients included in the current study.

	1. Multiplex IF-IHC (N = 62)		2. Serum IL-6 level (N = 16)	
	Characteristics	N (%)	Characteristics	N (%)
**Age**				
	≤ 65	34 (54.8)	≤ 65	8 (50.0)
	> 65	28 (45.2)	> 65	8 (50.0)
**Gender**				
	female	23 (37.1)	female	5 (31.3)
	male	39 (62.9)	male	11 (68.7)
**Type**[a]				
	ADC	49 (79.0)	ADC	11 (68.7)
	SCC	9 (14.5)	SCC	5 (31.3)
	LCC	4 (6.5)	LCC	0 (0.0)
**Stage**				
	stage I	21 (33.9)	stage I	0 (0.0)
	stage II	11 (17.7)	stage II	0 (0.0)
	stage III	22 (35.5)	stage III	1 (6.3)
	stage IV	8 (12.9)	stage IV	15 (93.7)
**T stage**				
	T1	12 (19.3)	T1	0 (0.0)
	T2	42 (67.7)	T2	2 (13.3)
	T3	4 (6.5)	T3	4 (26.7)
	T4	4 (6.5)	T4	9 (60.0)
**N stage**				
	N0	27 (43.6)	N0	1 (6.7)
	N1	10 (16.1)	N1	0 (0.0)
	N2	24 (38.7)	N2	5 (33.3)
	N3	1 (1.6)	N3	9 (60.0)
**M stage**				
	M0	53 (85.5)	M0	2 (13.3)
	M ≥ 1	9 (14.5)	M ≥ 1	13 (86.7)

[a]Abbreviation: ADC, adenocarcinoma; SCC, squamous cell carcinoma; LCC, large cell carcinoma
